# Novel interaction between neurotrophic factor-α1/carboxypeptidase E and serotonin receptor, 5-HTR1E, protects human neurons against oxidative/neuroexcitotoxic stress via β-arrestin/ERK signaling

**DOI:** 10.1007/s00018-021-04021-3

**Published:** 2021-12-29

**Authors:** Vinay Kumar Sharma, Xuyu Yang, Soo-Kyung Kim, Amirhossein Mafi, Daniel Saiz-Sanchez, Patricia Villanueva-Anguita, Lan Xiao, Leila Toulabi, Asuka Inoue, William A. Goddard, Y. Peng Loh

**Affiliations:** 1 Section on Cellular Neurobiology, Eunice Kennedy Shriver National Institute of Child Health and Human Development, National Institutes of Health, 49, Convent Drive, Bldg 49, Rm 6A-10, Bethesda, MD 20892 USA; 2 Materials and Process Simulation Center, California Institute of Technology, Pasedena, CA 91125 USA; 3 Neuroplasticity and Neurodegeneration Laboratory, Medical School, Regional Center for Biomedical Research, University of Castilla-La Mancha, 13071 Ciudad Real, Spain; 4 Graduate School of Pharmaceutical Sciences, Tohoku University, Sendai, Miyagi 980-8578 Japan

**Keywords:** GPCR, β-arrestin, Cytotoxicity, Neuroprotection

## Abstract

Protecting neurons from death during oxidative and neuroexcitotoxic stress is key for preventing cognitive dysfunction. We uncovered a novel neuroprotective mechanism involving interaction between neurotrophic factor-α1 (NF-α1/carboxypeptidase E, CPE) and human 5-HTR1E, a G protein-coupled serotonin receptor with no previously known neurological function. Co-immunoprecipitation and pull-down assays confirmed interaction between NFα1/CPE and 5-HTR1E and ^125^I NF-α1/CPE-binding studies demonstrated saturable, high-affinity binding to 5-HTR1E in stably transfected HEK293 cells (Kd = 13.82 nM). Treatment of 5-HTR1E stable cells with NF-α1/CPE increased pERK 1/2 and pCREB levels which prevented a decrease in pro-survival protein, BCL2, during H_2_O_2_-induced oxidative stress. Cell survival assay in β-arrestin Knockout HEK293 cells showed that the NF-α1/CPE-5-HTR1E-mediated protection against oxidative stress was β-arrestin-dependent. Molecular dynamics studies revealed that NF-α1/CPE interacts with 5-HTR1E via 3 salt bridges, stabilized by several hydrogen bonds, independent of the serotonin pocket. Furthermore, after phosphorylating the C-terminal tail and intracellular loop 3 (ICL3) of NF-α1/CPE-5-HTR1E, it recruited β-arrestin1 by forming numerous salt bridges and hydrogen bonds to ICL2 and ICL3, leading to activation of β-arrestin1. Immunofluorescence studies showed 5-HTR1E and NF-α1/CPE are highly expressed and co-localized on cell surface of human hippocampal neurons. Importantly, knock-down of 5-HTR1E in human primary neurons diminished the NF-α1/CPE-mediated protection of these neurons against oxidative stress and glutamate neurotoxicity-induced cell death. Thus, NF-α1/CPE uniquely interacts with serotonin receptor 5-HTR1E to activate the β-arrestin/ERK/CREB/BCL2 pathway to mediate stress-induced neuroprotection.

**Supplementary Information:**

The online version contains supplementary material available at 10.1007/s00018-021-04021-3.

## Introduction

Understanding mechanisms that can protect against neuronal cell death caused by various types of stress, such as neuroexcitotoxicity and oxidative stress, in the brain will facilitate the development of therapeutics that can mitigate these challenges. Indeed, many growth and trophic factors, such as transforming growth factors (TGFs), insulin-like growth factor (IGFs), epidermal growth factor (EGF), fibroblast growth factor 2 (FGF2) and brain-derived neurotrophic factor (BDNF), are known to play a role in neuroprotection [1–3]. Recently, we have identified a new trophin, neurotrophic factor-α1 (NF-α1), which is critical in protecting hippocampal neurons against stress-induced cell death in mice [4]. Interestingly, NF-α1 was originally characterized as carboxypeptidase E (CPE), a pro-hormone-processing enzyme, [5, 6], but our studies demonstrated that its multiple trophic functions are independent of its enzyme activity [4, 7]. NF-α1/CPE knock-out (KO) mice showed complete degeneration of hippocampal CA3 neurons, and cognitive dysfunction after emotional and physical stress, despite the expression of normal levels of BDNF and other growth factors in the hippocampus [4, 8]. In other studies, rats subjected to global ischemia revealed sustained increase in NF-α1/CPE expression in the hippocampal CA3 neurons, facilitating the survival of these neurons [9]. A mouse model of a human *cpe* gene mutation found in an Alzheimer’s disease (AD) patient showed hippocampal neuronal degeneration and memory loss [10]. Moreover, genetic studies identified null and non-sense mutations in the *cpe* gene in five families with a total of 8 members having homozygous mutations, presented clinical features of obesity, diabetes and learning disabilities [11–13]. Thus, NF-α1/CPE plays important trophic roles in neuronal cell survival and cognitive function in vivo [7].

Cell biological studies provided additional evidence that NF-α1/CPE promotes cell survival. NF-α1/CPE applied extracellularly protected rat hippocampal primary neurons against H_2_O_2_-induced cytotoxicity by activating ERK and AKT signaling cascades, which increased pro-survival protein, BCL2 and decreased caspase-3 activation [14, 15]. In murine skeletal stem cells, treatment with NF-α1/CPE activated ERK signaling and increased proliferation [16]. However, the mechanism of action of extracellular NF-α1/CPE in neuroprotection remains elusive.

We hypothesized that NF-α1/CPE action is mediated via interaction with a cell surface receptor, and employed a high-throughput screening strategy using NF-α1/CPE as a ligand against a library of 324 human G protein-coupled receptors (GPCRs) to identify a receptor. Only one of them, 5-hydroxytryptamine receptor 1-E (5-HTR1E), a Gi-coupled receptor showed a strong positive signal. 5-HTR1E is a member of the serotonin receptor family which is located to human chromosome 6q14-q15 [17] and is expressed in humans, monkeys and guinea pig, but not in rats or mice [18–21]. Pharmacological and immunocytochemical localization studies in the guinea pig brain indicate that 5-HTR1E is abundant in the hippocampus, frontal cortex, and olfactory bulb, but with no known function in brain [21, 22]. Recently, Qin et al. [23] reported that 5-HTR1E signaling may prevent the chronic psychological stress-promoted progression of ovarian cancer and might have potential therapeutic value.

Here we carried out co-immunoprecipitation and pull-down studies to show interaction between NF-α1/CPE and 5-HTR1E, and radiolabeled NF-α1/CPE-binding experiments to determine the binding affinity to 5-HTR1E. We used molecular dynamics (MD) methods to predict the binding site of NF-α1/CPE to 5-HTR1E. Studies were also conducted on 5-HTR1E-transfected HEK293 cells as a model to determine the signal transduction cascade activated by the NF-α1/CPE-5-HTR1E interaction to mediate cell survival during oxidative stress. The co-expression and neuronal surface membrane co-localization of NF-α1/CPE and 5-HTR1E in human hippocampus was mapped by immunohistochemistry. Finally, the effect of 5-HTR1E knockdown in human primary neurons, on neuroprotection was investigated. Our experimental and computational findings indicate that NF-α1/CPE interacts with 5-HTR1E in a novel manner, independent of the serotonin pocket, to promote survival of neurons and other human cells via β-arrestin recruitment and activation of pERK-CREB-BCL2 signaling pathway.

## Materials and methods

### Cells

HTLA cells were purchased from Add gene, USA. HEK293 cells stably expressing 5-HTR1E were obtained from Dr. Bryan Roth’s laboratory at University of North Carolina (UNC). HEK293, U118 and LN18 glioblastoma cells were purchased from ATCC (Manassas, VA). Human brain primary neurons (cat. no. 1520) and extract (cat. no. 1526) were purchased from ScienCell Research Laboratories, Carlsbad, CA.

### Recombinant NF-α1/CPE

Recombinant mouse NF-α1/CPE (53 kDa) was custom-synthesized and highly purified by GenScript, NJ, USA [4]. Mouse NF-α1/CPE protein (uniport id:Q00493) sequence is 96.6% homologous to human (uniport id:P16870).

### DNA constructs

Plasmids expressing human wild type and truncated fragments of NF-α1/CPE (residues 1–150, 1–350, 351–476) with a C-terminal V5 tag were constructed into pcDNA 3.1 backbone by GenScript (GenScript, NJ, USA). Plasmid expressing human 5-HTR1E with a C-terminal Myc tag was from Origene (Rockville, MD).

### Western blot

Cells were lysed in RIPA (cat. No. 89901) or Pierce™ IP Lysis Buffer (cat. No. 87787), Thermo scientific, USA. Proteins were quantified using BCA or Bradford assay and separated on SDS-PAGE gel followed by western blotting. Protein bands were visualized and quantified by the Odyssey infrared imaging system and software (LI-COR Inc, Lincoln, NE).

### Luciferase assay for 5-HTR1E receptor

The PRESTO-Tango GPCR assay kit and HTLA cells were purchased from Addgene. This assay is based on the ‘transcriptional activation following arrestin translocation’ (TANGO). Upon activation by ligand molecules, GPCR recruits β-arrestin2-TEV fusion protein (stably expressing in HTLA cells) to the V2 tail which is present in the same plasmid followed by a TEV cleavage site and tTA transcription factor. Cleavage at the GPCR–TEV protein site releases the tTA, which, after transport to the nucleus, activates tTA-dependent luciferase reporter gene (see Suppl. Fig. S1) [23]. HTLA cells (10,000 cells/well) were plated in a white 96-well plate in DMEM supplemented with 10% FBS. On the next day, 0.2 μg/well 5-HTR1E or control plasmid was transfected in serum-free media. Renila luciferase was also transfected as internal control. After 48 h of transfection, cells were treated with different doses of recombinant NF-α1/CPE (0–100 nM) or 5-HT (0–1 μM) for 3 h. Luciferase assay was performed using Dual-Luciferase® Reporter Assay System (Cat. No. E1910, Promega, USA), according to manufacturer’s protocols. Luminescence was measured using a Synergy HTX plate reader (Biotech, USA).

### Radioligand-binding studies

For binding experiments, custom-labeled [^125^I] NF-α1/CPE (PerkinElmer, USA) was used to bind 5-HTR1E stable or control HEK293 cells. These cells (~ 200,000) were collected in 5 ml polystyrene tubes and washed three times with serum-free binding medium (DMEM) and incubated with different concentrations (1.25–30 nM, triplicates for each data point) of [^125^I] NF-α1/CPE (hot) in 1 ml serum-free binding medium for 3 h. on ice. 500 nM unlabeled NF-α1/CPE protein (cold) or carboxypeptidase B (porcine pancreas CPB, Sigma Aldrich, USA) was also incubated for non-specific binding and displacement experiments. After incubation, cells were washed four times with cold PBS (2 ml each time) and counted in a Perkin Elmer gamma-counter. For displacement and specificity experiments, cells were incubated with 30 nM [^125^I] NF-α1/CPE in the presence and absence of 500 nM cold NF-α1/CPE or CPB, (a negative control protein for NF-α1/CPE).

### Co-immunoprecipitation

NF-α1/CPE-positive LN-18 cells were lysed in immunoprecipitation lysis buffer (50 mmol/L Tris–HCL, 150 mmol/L NaCl, 5 mmol/L EDTA, 0.5% NP-40) supplemented with proteinase inhibitor cocktail (Sigma-Aldrich) and cleared by centrifugation at 12,000 × *g* for 20 min at 4°C. A total of 1 mg protein of precleared cell lysates was incubated with 4 μg of anti-5-HTR1E polyclonal antibodies (Abcam) or anti-NF-α1/CPE monoclonal antibody (BD Bioscience) and rotated at 4°C overnight. The immuno-complexes were recovered by incubation with 30 μl of 50% of protein A/G-Sepharose slurry (Santa Cruz) at 4°C overnight. The pellets were washed 4 times with IP lysis buffer and the bound proteins were eluted in loading buffer at 65°C for 5 min. and subjected to immunoblotting analyses using an anti-NF-α1/CPE or anti-5-HTR1E antibody.

### Pull-down assay

A series of vectors for expressing NF-α1/CPE and truncated fragments of NF-α1/CPE (residues 1–150, 1–350, 351–476) with a C-terminal V5 tag were constructed into pcDNA3.1 vector for pull-down assay. Unlike the other two truncated fragments CPE1–150 and CPE 1–350, truncated fragment CPE 351–476 has no signal peptide. All constructs were verified by DNA sequencing. For pull-down assay, HEK293 cells were co-transfected with a combination of plasmids expressing 5-HTR1E-myc (Origene), and CPE or its derivatives with V5 tag using Lipofectamine 2000 for 48 h. Transfected cells were harvested and lysed with immunoprecipitation lysis buffer. 1 mg protein of each total cell lysate was incubated with 20 μl of anti-Myc or anti-V5-conjugated agarose (Santa Cruz) overnight at 4°C. Pellets were washed 4 times with lysis buffer and the bound proteins were eluted in LDS loading buffer (Invitrogen) at 65°C for 5 min. and subjected to immunoblotting analysis with anti-V5 antibody (Invitrogen) or anti-Myc antibody (Santa Cruz).

### Molecular docking of NF-α1/CPE with 5-HTR1E

In order to predict which NF-α1/CPE residues bind to 5-HTR1E surface residues, both NF-α1/CPE and 5-HTR1E structures were predicted using available templates in the PDB database. We then used these structures to predict the binding site for CPE. The detailed methodology is in the Supplementary methods S1.

### Molecular dynamics simulation of β-arrestin with 5-HTR1E

For detailed methodology, see Supplementary methods S2.

### ERK phosphorylation

5-HTR1E stable and HEK293 control cells were seeded in 12-well plate (1 × 10^5^/well) in complete DMEM media and incubated overnight at 37°C in a CO_2_ incubator. β-arrestin knockout cells were generated and characterized as previously described (2) and control HEK293 cells were transfected with 5-HTR1E plasmid for 48 h. On the next day, media were changed to serum-free medium (SFM) and after 3 h, cells were treated with different concentrations (0–50 nM) of NF-α1/CPE or (0–1 μM) 5-HT for 5–10 min. For inhibitor studies, control and 5-HTR1E-expressing HEK293 cells were treated with 1 μm FR900359 for 30 min or 200 ng PTX for 4 h prior to treatment with 50 nM NF-α1. Change in pERK was assessed by probing the western blot membranes with pERK1/2 (Thr202/Tyr204) rabbit antibody (1:5000, Cell signaling, Danvers, MA) and tERK1/2 mouse monoclonal antibody (1:5000, Cell signaling, Danvers, MA) simultaneously. Fluorescence-labeled anti-rabbit (800 nm) and anti-mouse (680 nm) secondary antibodies (1:5000) were used to visualize the protein bands. pERK was normalized with tERK, and fold changes were calculated from three independent experiments after densitometric analysis using image J software, NIH.

### CREB phosphorylation

5-HTR1E stable cells were seeded in 12-well plate (1 × 10^5^/well) in complete DMEM. On the next day, after media were changed to SFM for 3 h, cells were treated with 50 nM NF-α1/CPE at different time points (10–60 min). For the control experiments, HEK293 cells were also treated with 50 nM NF-α1/CPE. Effect of 5-HT on CREB phosphorylation was analyzed by treating 5-HTR1E stable cells with 1 μM 5-HT in the presence and absence of 10 μM forskolin. Changes in pCREB levels were assessed by Western blot using pCREB rabbit mAb against Ser133 (Cat. No. #9198, Cell signaling, Danvers, MA) and tCREB mouse mAb (Cat. No. #9104, Cell signaling, Danvers, MA) for loading control.

### cAMP assay

cAMP assay was performed in 5-HTR1E stable cells. 5000 cells/well were seeded in a 96-well plate in complete DMEM overnight. On the next day, media were replaced with SFM for 3 h. These cells were treated with 10 μM forskolin (an activator of adenylyl cyclase enzyme to raise cAMP levels) in the presence of phosphodiesterase inhibitor (Cat. no. 524718, set I-Calbiochem, Sigma Aldrich, USA) for 10 min followed by 1 μM 5-HT, BRL54443 (5-HTR1E/F agonist), or 50 nM NF-α1/CPE for 20 min. To check the synergistic effect, 5-HTR1E-expressing cells were also treated with a combination of 1 μM 5-HT and 50 nM NF-α1/CPE. cAMP assay was performed using cAMP-Glo™ Assay (Promega, USA) according to manufacturer’s instructions. Luminescence was recorded on plate reader. All experiments were performed in triplicate and repeated at least three times.

### BCL2 protein expression

5-HTR1E stable cells and HEK293 control cells were seeded in a 12-well plate. A day later, cells were treated with 50 nM recombinant NF-α1/CPE protein or 1 μM 5-HT in serum-free medium for 6 h followed by 200 µM H_2_O_2_ for overnight. BCL2 expression was analyzed by western blot using BCL2 antibody (rabbit, Cell signaling, USA).

### Lactate dehydrogenase assay

HEK293 cells were transfected with 5-HTR1E plasmid or empty control vector for 48 h using Lipofectamine 2000 reagent kit (Invitrogen, Carlsbad, CA). While β-arrestin KO and their control HEK293 cells (10,000 cells per well) were infected with Ad-5-HTR1E or control Ad-LacZ (Vector Biolabs) at 10 MOI, for 48 h. These cells were treated with 50 nM CPE overnight followed by 300 µM H_2_O_2_ for 6 h. Human primary neurons were seeded in 96-well, poly-d-lysine-coated plates at a density of 13,000 neurons/well in neuronal medium (Cat. #1521, ScienCell Research Laboratories, Carlsbad, CA) and cultured until they were attached to the plate. The neurons were then infected with Ad-5-HTR1E shRNA or Ad-LacZ (Vector Biolabs) at 30 MOI, for 72 h and then overnight treatment with 50 nM CPE. The medium was replaced with neuronal medium without growth factors and the neurons challenged with 100 µM H_2_O_2_ for 6 h or 40 µM glutamate (Sigma-Aldrich, St. Louis, MO) for 24 h. Cytotoxicity was measured by the amount of LDH released using a CytoTox 96 assay kit (Promega, USA).

### Immunofluorescence and immunohistochemistry of human hippocampus

Distribution of 5-HTR1E and NF-α1/CPE in the hippocampus of six human postmortem brain tissues (without neuropathology) was assessed by immunofluorescence and immunohistochemistry experiments. Brain tissues were obtained from three national brain banks, Barcelona IDIBAPS, Murcia BIOBANC-MUR and Madrid BTCIEN, Spain. For detailed methods, see supplementary methods S3.

### Statistical analysis

Data are representative of at least 3 separate experiments (N), with each experiment performed in triplicate. Data were analyzed by 2-tailed Student’s *t* test or 1-way ANOVA, followed by Tukey’s post hoc multiple comparisons tests, were noted. Statistical significance was defined as *p* < 0.05.

## Results

### NF-α1/CPE interacts with 5-HTR1E serotonin receptor

**Fig. 1:**
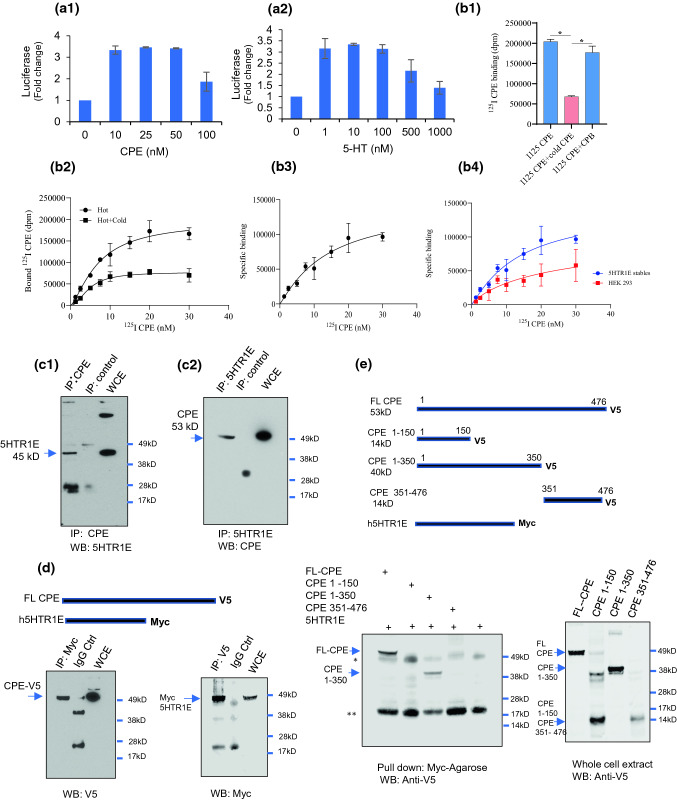
**a** Luciferase assay. HTLA cells were transfected with 5-HTR1E plasmid for 48 h and dose dependent effect of **a1** CPE and **a2** 5-HT (serotonin) were checked by dual luciferase assay after 3 h of treatment. Results are expressed as fold change, values are mean ± SD, *n* = 3, *N* = 2. CPE binds to 5-HTR1E on the cell surface. **b** ^125^I CPE binding to 5-HTR1E stable cells. **b1** 5-HTR1E stable cells were incubated with 30 nM ^125^I radiolabeled CPE (hot) for 3 h on ice in serum-free binding medium. To demonstrate binding specificity, 500 nM non-radio-labeled CPE (cold) or CPB was co-incubated with the hot CPE to compete and displace the hot CPE binding. The bar graphs show that the ^125^I CPE binding to 5-HTR1E was displaced by cold CPE but not by CPB. One-way ANOVA analysis followed by Tukey’s post hoc multiple comparison test, [*F* (2, 6) = 169, *p* < 0.0001], For ^125^I CPE + cold CPE compared to ^125^I CPE, *p < 0.0001, ^125^I CPE + cold CPE compared to ^125^I CPE + cold CPB, *p < 0.0001, values are mean ± SD, *n* = 3. **b2–3** ^125^I CPE binds to 5-HTR1E in a saturable manner. 5-HTR1E stable cells were incubated with different concentrations (1.25–30 nM) of ^125^I CPE (hot) with or without 500 nM cold CPE for 3 h on ice in serum-free binding medium. The specific binding and K_d_ were determined by measuring bound ^125^I CPE in the presence of 500 nM cold CPE. (B4) Comparison of ^125^I CPE binding between 5-HTR1E stable and control HEK293 cells. **c **Co-immunoprecipitation experiments. Extracts from LN-18 cells were immunoprecipitated (IP) using 5-HTR1E or CPE antibodies followed by immunoblotting with anti-CPE **(c1)** or anti-5-HTR1E **(c2**); the arrowhead indicates co-precipitated CPE and 5-HTR1E protein bands. Total cell lysate (WCE) was used as positive control, normal rabbit or mouse serum (IP Ctrl) was used as IP control. **d** Pull-down experiments. HEK293 cells were co-transfected with V5-CPE and Myc-5-HTR1E. Pull-down assays were carried out by incubating cell extracts with anti-V5 and anti-Myc-conjugated agarose overnight and pellets were analyzed by immunoblotting with anti-V5 antibody or anti-Myc antibody, respectively. Left panel: pull-down with anti-myc and probed with anti-V5, arrow shows CPE-V5; Right panel: pull-down with anti-V5 and probed with anti-myc, arrow shows myc-5-HTR1E. **e** Analysis of CPE domains binding to 5-HTR1E. Top: schematic representation of constructs expressing CPE and its derivatives with V5 tag and 5-HTR1E with Myc tag. Middle, full-length CPE (1–476) or truncated CPE fragments with V5 tag was co-transfected with 5-HTR1E with Myc tag in HEK293 cells, transfected cells were harvested and lysed. Pull-down assay was carried out by incubating cell extracts with Myc-conjugated agarose, and pellets were analyzed by immunoblotting with anti-V5 antibody. Bottom: expression of CPE and its derivatives in transiently transfected HEK293 cells. Note that only CPE-1-350 was pulled down

High-throughput screening of NF-α1/CPE against a GPCR library was performed using the β-arrestin biased PRESTO-Tango assay, discovering 5-HTR1E as a potential target receptor for further study (Suppl. Fig. S1) [24]. To verify that NF-α1/CPE binds 5-HTR1E, HTLA cells expressing 5-HTR1E receptor were treated with different doses of recombinant NF-α1/CPE or its known ligand serotonin. NF-α1/CPE activated 5-HTR1E in the luciferase assay (see methods) within 3 h of treatment and optimally with 10–25 nM doses (Fig. 1a1) while serotonin (5-HT) saturated the receptor between 1 and 10 nM concentration (Fig. 1a2).

To determine if NF-α1/CPE binds to 5-HTR1E on the cell surface membrane, we performed radioligand-binding assays. 5-HTR1E stable cells were incubated with [^125^I] NF-α1/CPE (hot), with or without non-radiolabeled NF-α1/CPE (cold), to compete with the hot NF-α1/CPE binding. A decrease in total bound ^125^I NF-α1/CPE was observed in the presence of cold NF-α1/CPE, showing competition and specific binding of hot NF-α1/CPE (Fig. 1b1), while there was no displacement in total [^125^I] NF-α1/CPE bound when Carboxypeptidase B (CPB) was added with [^125^I] NF-α1/CPE, verifying specificity of the binding. Saturation-binding experiments were also performed using various concentrations of [^125^I] NF-α1/CPE (Fig. 1b2) with and without cold NF-α1/CPE. Specific binding was obtained by subtracting non-specific binding from total binding (Fig. 1b3). Saturation-binding data were analyzed by non-linear regression analysis using the GraphPad PRISM 8.0 program. The best fit Kd value was 13.82 nM for ^125^I NF-α1/CPE. Similar experiments were performed in control HEK293 cells. A small amount of other cell surface binding was observed (Fig. 1b4 and Suppl. Fig. S2). These results indicate that NF-α1/CPE binds specifically and with high affinity to 5-HTR1E on cell surface.

### NF-α1/CPE interacts with 5-HTR1E via specific molecular domains

To verify that NF-α1/CPE-5-HTR1E interaction occurs with these endogenous proteins in mammalian cells, we carried out co-immunoprecipitation experiments in a human glioblastoma cell line, LN-18, that expresses high levels of endogenous 5-HTR1E protein. Interaction between NF-α1/CPE and 5-HTR1E proteins was detected in these LN-18 cell extracts, (Fig. 1c1 and c2). To demonstrate interactions between NF-α1/CPE and 5-HTR1E and then to identify the NF-α1/CPE-binding domains required for NF-α1/CPE-5-HTR1E interaction, we constructed a series of vectors expressing full-length and truncated fragments of NF-α1/CPE (residues 1–150, 1–350, 351–476) with a C-terminal V5 tag and 5-HTR1E-myc, which were transfected into HEK293 cells for pull-down assays. All the fragments were well expressed and detected in the lysates (Fig. 1e). Full-length NF-α1/CPE (Fig. 1d), as well as NF-α1/CPE fragment 1–350 (Fig. 1e) showed strong binding to 5-HTR1E, whereas binding of the other fragments (1–150, 351–476, Fig. 1e) was not detected. The results indicate that the middle region of NF-α1/CPE (amino acids 150–350) harbors a binding motif(s) for 5-HTR1E (Fig. 1e).

### Molecular docking and molecular dynamics studies predict strong interactions between NF-α1/CPE and the extracellular loops of 5-HTR1E

To predict the molecular interaction of NF-α1/CPE with 5-HTR1E, we first generated the 3D structure of NF-α1/CPE using homology modeling. To predict the transmembrane domain structure of 5-HTR1E, we applied our GEnSeMBLE complete sampling procedure to predict the pre-activated conformation of 5-HTR1E, and then we added loops using a homology strategy. Leveraging our biochemical binding assays in Fig. 1e, we used our DarwinDock techniques and molecular dynamics (MD) to construct initial structures for the interaction between NF-α1/CPE (residues 151–350) and 5-HTR1E (extracellular portion). See supplementary information (SI, methods section, results SK1-SK2 and Fig. SK1–SK4). Fig. SK4 shows the total, electrostatic and van der Waals (vdW) interaction energies of 5-HTR1E and NF-α1/CPE through 100 ns MD trajectories. Until 5 ns, 4 SBs in the complex were constrained. After 5 ns, the structure was relaxed without any constraints. The initial minimized structure of 5-HTR1E and NF-α1/CPE complex shows interactions between the positive-charged residues in the ECL and negative-charged residues in NF-α1/CPE.

**Fig. 2:**
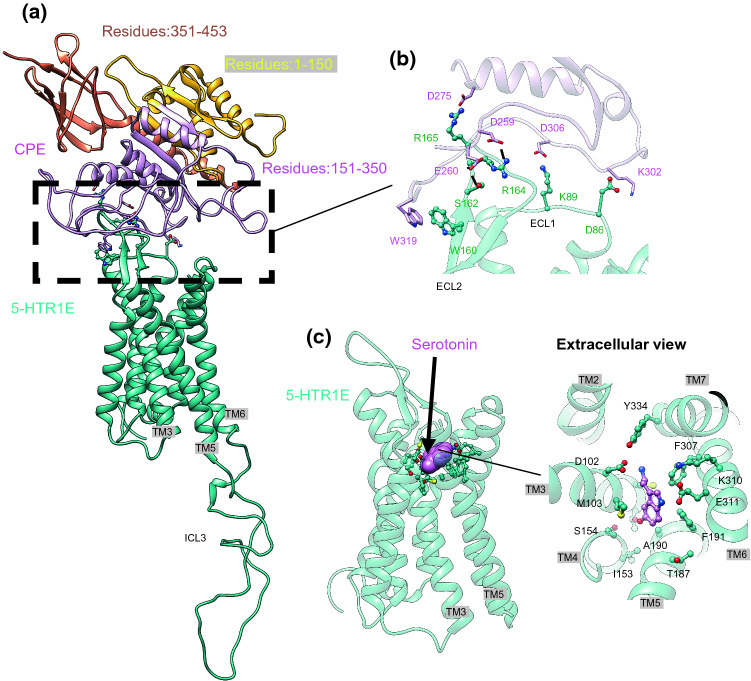
High-affinity CPE-5-HTR1E coupling. **a** The side view of binding interface between CPE and 5-HTR1E in the presence of β-arrestin1 (omitted for clarity) resulted from ~ 1.5 μs MD simulations colored by subunits: CPE (pink), 5-HTR1E (green). **b** Tight coupling between the CPE and the extracellular surface of 5-HTR1E, mostly involving the polar interactions between the pair proteins. **c** The orthosteric serotonin-binding pocket was stabilized through salt bridge between the protonated nitrogen and D102 (3.32) and H-bonding between NH and E311 (6.55)

This was followed by 1.5 µs of MD which led to the strong salt bridge (SB) interactions between 5-HTR1E and NF-α1/CPE shown in Fig. 2. Our MD simulations show that the NF-α1/CPE couples strongly to the extracellular loops (ECL) of 5-HTR1E (Fig. 2a), which may block the path for other small molecules to penetrate the orthosteric binding pocket. Our MD studies find that NF-α1/CPE forms polar interactions with the extracellular portion of 5-HTR1E (Fig. 2b). In particular, salt bridges: K302-D86^ECL1^, D306-K89^ECL1^, D275-R165^ECL2^, hydrogen bonds: D259- R164^ECL2^, E260-S162^ECL2^, and an aromatic interaction between W319-W160^ECL2^ participate in strong coupling between NF-α1/CPE and 5-HTR1E. We found that the orthosteric serotonin-binding pocket of 5-HTR1E was stabilized through a salt bridge between the protonated nitrogen and D102^3.32^ and an H bond between NH and E311^6.55^, as shown in Fig. 2c.

Our MD studies find that binding NF-α1/CPE stabilizes the active-state conformation of 5-HTR1E, featuring an expanded intracellular region (Fig. 5e). The outward movement of the cytosolic end of TM6 from TM3 is the hallmark of class A GPCR activation [25]. However, a polar lock between conserved residues R120^3.50^ [the superscript is Ballesteros–Weinstein numbering for GPCRs [26], taken from Ref. [27] and E268^6.30^ in the inactive-state serotonin receptor [28], impedes TM6 from the outward displacement associated with normal G protein activation. Disruption of this polar lock is crucial for GPCR activation [29–31] and subsequent recruitment of downstream effectors. To examine if NF-α1/CPE binding stabilizes the active-state conformation of 5-HTR1E, we examined the distance between TM3 and TM6 (Fig. 5c). Our analysis indicates that the cytoplasmic region of 5-HTR1E remains fully open with an average distance of ~ 18.1 ± 0.1 Å [measuring the distance between R120^3.50^ (CZ)- E268^6.30^(CD)], compared to ~ 6 Å for the crystallographic inactive 5-HT_2A_ receptor [28].

### 5-HTR1E-NF-α1/CPE interaction activates ERK/CREB pathway

**Fig. 3:**
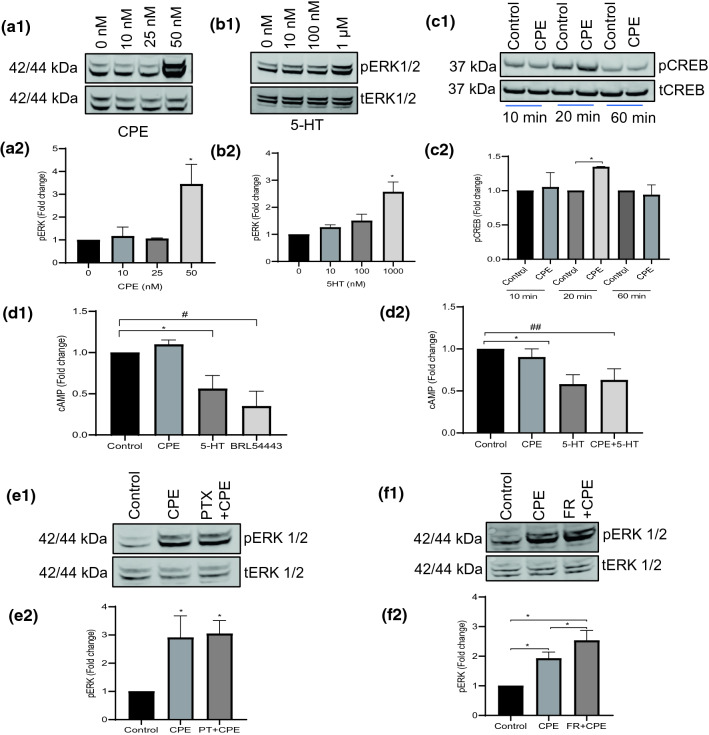
**a**–**c** CPE activates ERK signaling in HEK293 cells via interaction with 5-HTR1E. HEK293 cells stably transfected with 5-HTR1E were treated with 0–50 nM CPE **(a1–2)** or 0 nM to 1 μM 5-HT **(b1–2)** for 5–15 min and pERK 1/2 were analyzed by Western blotting. Bar graphs showing the fold change in pERK1/2 after normalization with tERK1/2 as an internal control. *N* = 3, One-way ANOVA analysis followed by Tukey’s post hoc multiple comparison test, [*F* (3, 8) = 18.60, *p = 0.0006], *p = 0.0011 for 50 nM CPE when compared to untreated control and [*F* (3, 8) = 28.18, *p = 0.0001], *p = 0.0001 for 1 µM 5-HT when compared to untreated control, values are mean ± SD, *N* = 3. HEK293 control cells were also treated with CPE or 5-HT. One-way ANOVA analysis followed by Tukey’s post hoc multiple comparison test, *p* > 0.05 (ns), *N* = 3 (Suppl. Fig. 3A–B). **c** CPE increases CREB phosphorylation in 5-HTR1E stable cells. 5-HTR1E stable cells **(c1–2)** and control HEK293 cells (Suppl. Fig. 3, c1–2) were treated with 50 nM CPE for different time points (between 10 and 60 min) and analyzed by Western blotting. Bar graphs showing the fold change in pCREB after normalization with tCREB as an internal control. Student’s *t *test ***p < 0.0001 for 50 nM CPE when compared to untreated control, values are mean ± SD, *N* = 3 (**d**). CPE does not affect forskolin-stimulated cAMP. 5-HTR1E stable cells (pretreated with 10 μM forskolin for 10 min) were treated with 50 nM CPE, 1 μM 5-HT, 5-HTR1E agonist BRL 54,443 **(d1)**, or both 50 nM CPE and 1 μM 5-HT **(d2)** simultaneously for 20 min, and changes in cAMP levels were measured by cAMP-Glo™ Assay kit. One-way ANOVA analysis followed by Tukey’s post hoc multiple comparison test, [*F* (3, 8) = 24.7, *p* = 0.0002], CPE *p* = ns, 5-HT *p = 0.0109, BRL 54,443 *p = 0.0009, CPE + 5-HT *p < 0.0001, compared to control, values are mean ± SD, *N* = 3 **(e–f)**. CPE-activated ERK phosphorylation is independent of G proteins. 5-HTR1E stable cells were treated with 200 ng PTX (Gi inhibitor) for 4 h or 1 μm FR900359 (Gq inhibitor) for 30 min followed by 50 nM CPE **(e1–2**; **f1–2)** and pERK 1/2 were analyzed by Western blotting. Bar graphs showing the fold change in pERK1/2 after normalization with tERK1/2 as an internal control. One-way ANOVA analysis followed by Tukey’s post hoc multiple comparison test. For **e1–2** [*F* (2, 6) = 14.53, *p* = 0.005], control vs CPE *p = 0.0097, control *vs* CPE + PTX *p = 0.0070, *N* = 3. For **f1–2** [*F* (2, 6) = 33.90, *p* = 0.0005], control *vs* CPE *p = 0.0062, control vs CPE + FR900359 *p = 0.0004, values are mean ± SD, *N* = 3

NF-α1/CPE has been shown to activate ERK signaling in hippocampal neurons [14]. To determine if NF-α1/CPE activates the ERK pathway via 5-HTR1E, we treated 5-HTR1E stable or control HEK293 cells with 0–50 nM NF-α1/CPE for 5–15 min and assayed for ERK phosphorylation. A significant increase (3.4-fold) in phosphorylation of ERK 1/2 was observed in 5-HTR1E stable cells treated with 50 nM NF-α1/CPE between 5 and 10 min (Fig. 3a), but not in control, HEK293 cells (Suppl. Fig. S3, panel a1–2). To determine if serotonin could also activate the ERK signaling pathway via 5-HTR1E receptor, we treated 5-HTR1E stable cells with 0 nM to 1 μM 5-HT for 5–15 min. We found that at a high dose of 1 μM, 5-HT increased ERK 1/2 phosphorylation 2.5-fold (Fig. 3b). Control HEK293 cells were also treated with 0–1 μM 5-HT in similar manner but did not show any significant increase in ERK1/2 phosphorylation (Suppl. Fig. S3, panel b1–2).

Since CREB is a downstream effector of ERK signaling pathways and its phosphorylation is positively related to cell survival against oxidative stress and regulation of pro-survival protein BCL2 [32, 33], we analyzed the effect of NF-α1/CPE on CREB phosphorylation. 5-HTR1E stable or control HEK293 cells were treated with 50 nM NF-α1/CPE and change in pCREB was assessed by Western blotting. After 20 min of treatment, an increase (1.3-fold) in CREB phosphorylation was observed in 5-HTR1E stable cells (Fig. 3d) but not in control HEK293 cells (Suppl. Fig. S3, panel c1–2). These results show that NF-α1/CPE can increase CREB phosphorylation via 5-HTR1E interaction.

### 5-HTR1E-NF-α1/CPE interaction does not inhibit cAMP pathway

5-HTR1E is a Gi-linked receptor that reduces cyclic AMP via inhibition of adenyl cyclase. To determine the effect of NF-α1/CPE alone and in combination with 5-HT on cAMP, we treated 5-HTR1E stable cells with 50 nM NF-α1/CPE or 1 μM 5-HT or both, in the presence of forskolin. Our results show that NF-α1/CPE-treated cells alone or in combination with 5-HT did not affect cAMP levels, while 5-HT and BRL 54,443, a 5-HTR1E agonist reduced the level of cAMP 44% and 66%, respectively (Fig. 3d1, d2). These results indicate that NF-α1/CPE-5-HTR1E function is not linked to the classical 5-HTR1E signaling mechanism which maintains an efficient Gi protein-coupling profile for 5-HT-induced cyclic AMP signaling.

### 5-HTR1E-mediated ERK signaling is independent of Gq or Gi proteins

To further explore whether the mechanism through which NF-α1/CPE activates ERK phosphorylation in 5-HTR1E-expressing cells involves Gi proteins, we tested the effect of Gi inhibitor PTX and Gq inhibitor FR900359 [34] on NF-α1-activated ERK phosphorylation. After 4 h incubation with or without PTX, cells were treated with 50 nM NF-α1 and compared with cells not treated with PTX. Figure 3, panel e1–2 shows that there was no significant difference in pERK1/2 phosphorylation between PTX-treated and -untreated groups. Figure 3f1–2 shows that after treatment with 50 nM NF-α1, pERK1/2 level increased slightly in FR900359-pretreated cells. These results show that Gi inhibitor PTX and Gq inhibitor FR900359 did not inhibit NF-α1 activation of ERK phosphorylation.

### 5-HTR1E mediates ERK phosphorylation and cell survival via β-arrestin recruitment

**Fig. 4:**
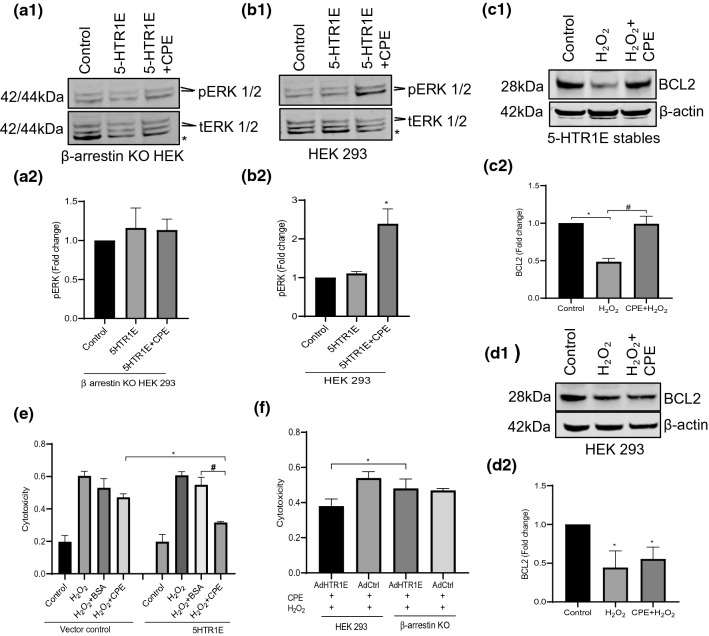
**a**, **b** CPE-5-HTR1E-induced ERK is activated through β-arrestin. 5-HTR1E expressing β-arrestin KO HEK293 **(a)** and control **(b)** cells were treated with 50 nM CPE and pERK was analyzed by Western blotting. Bar graphs showing the fold change in pERK1/2 after normalization with tERK1/2 as an internal control. One-way ANOVA analysis followed by Tukey’s post hoc multiple comparison test, **a** For β-arrestin KO HEK293 [*F* (2, 6) = 0.7779, *ns*] and **b** for control HEK293 [*F* (2, 6) = 35.33, *p* = 0.0005], control vs 5-HTR1E *p* = *ns*, control vs 5-HTR1E + 5-HT *p = 0.0007, values are mean ± SD, *N* = 3. (*) non-specific band (**c–d)**. CPE protects against H_2_O_2_-induced oxidative stress via 5-HTR1E- β arrestin. Western blot analysis of BCL2 protein in 5-HTR1E stable cells treated with 50 nM CPE **(c1–2)** for 6 h followed by 200 μM H_2_O_2_ treatment overnight. Bar graphs showing quantification of BCL2 protein after normalization with β-actin. Results are expressed as fold change, One-way ANOVA analysis followed by Tukey’s post hoc multiple comparison test, [*F* (2, 6) = 63.70, *p* < 0.0001], controls vs H_2_O_2_ *p = 0.0002, controls vs H_2_O_2_ + CPE *p* = *ns*, H_2_O_2_ vs H_2_O_2_ + CPE #*p* = 0.0002, values are mean ± SD, *N* = 3, **(d1–2)** HEK293 cells devoid of 5-HTR1E receptor were also analyzed for BCL2, [*F* (2, 6) = 11.09, *p* = 0.0096], controls vs H_2_O_2_ *p = 0.0103, controls vs H_2_O_2_ + CPE *p* = 0.0276, H_2_O_2_ vs H_2_O_2_ + CPE *p = 0.6658, value are mean ± SD, *N* = 3. Lactate dehydrogenase (LDH) cytotoxicity assay **(e)** HEK 293 cells were transfected with 5-HTR1E or pCDNA 3.1 plasmid for 48 h. Cells were treated with 50 nM CPE or BSA followed by 300 μM H_2_O_2_ for 6 h. One-way ANOVA analysis followed by Tukey’s post hoc multiple comparison test [*F* (7,16) = 67.43, *p* < 0.0001] #*p* < 0.0001 for 5-HTR1E + H_2_O_2_ + CPE compared to 5-HTR1E + H_**2**_O_**2**_ + BSA; *p = 0.0016 for 5-HTR1E transfected cells + H_2_O_2_ + CPE compared to vector control cells + H_2_O_2_ + CPE, values are mean ± SD, *N* = 3. **f** Control and β-arrestin KO HEK293 cells were transduced with 5-HTR1E or control adenovirus for 48 h. Cells were then treated with 50 nM CPE followed by 300 μM H_**2**_O_**2**_ for 6 h. One-way ANOVA analysis followed by Tukey’s post hoc multiple comparison test for LDH release assay: [*F* (2,32) = 27, *p* < 0.0001] *p < 0.05 for 5-HTR1E + H_2_O_2_ + CPE in HEK293 cells compared to 5-HTR1E + H_**2**_O_**2**_ + CPE in β-arrestin KO HEK293 cells, values are mean ± SD, *N* = 3

For some GPCRs, ERK signal transduction pathway can be activated via β-arrestin recruitment. We therefore determined if NF-α1/CPE-induced ERK 1/2 phosphorylation by 5-HTR1E is dependent on β-arrestin. Figure 4b1–2 showed the increase in ERK 1/2 phosphorylation in 5-HTR1E-transfected HEK293 cells was abolished in 5-HTR1E-transfected β-arrestin KO HEK293 cells (Fig. 4a1–2). These data indicate that β-arrestin plays a role in 5-HTR1E-mediated ERK phosphorylation.

Previously, NF-α1/CPE was shown to up-regulate pro-survival BCL2 protein expression in hippocampal neurons with H_2_O_2_-induced oxidative stress [14]. Here we determined if such an effect can occur because of interaction between NF-α1/CPE and 5-HTR1E. 5-HTR1E stable cells were treated with 50 nM NF-α1/CPE for 6 h followed by 200 μM H_2_O_2_ treatment overnight.  ~ 52% reduction in the mitochondrial pro-survival BCL2 protein level was observed after H_2_O_2_ treatment in 5-HTR1E stable cells, which was prevented by NF-α1/CPE treatment (Fig. 4c1–2) but not by serotonin (Suppl. Fig S4). These experiments were also done in control HEK293 cells, but no effect was observed (Fig. 4d1–2). We then showed that pretreatment with NF-α1/CPE effectively decreased the H_2_O_2_-induced cytotoxicity in 5-HTR1E expressing cells compared with BSA control. In contrast, HEK293 cells transfected with vector control, pretreatment with NF-α1/CPE did not decrease cytotoxicity when challenged with H_2_O_2_ (Fig. 4e). To determine if β-arrestin recruitment is involved in 5-HTR1E-mediated protection against H_2_O_2_-induced cytotoxicity, the experiments were performed in 5-HTR1E expressing, control and β-arrestin KO HEK293 cells. Figure 4f shows that treatment with NF-α1/CPE did not protect 5-HTR1E expressing β-arrestin KO cells against H_2_O_2_-induced cytotoxicity, unlike 5-HTR1E-expressing HEK293 cells. Thus, the function of the NF-α1/CPE–5-HTR1E interacting complex in promoting cell survival during H_2_O_2_-induced oxidative stress requires recruitment of β-arrestin.

### Molecular dynamics studies predict the NF-α1/CPE–5-HTR1E complex activates β-arrestin

**Fig. 5:**
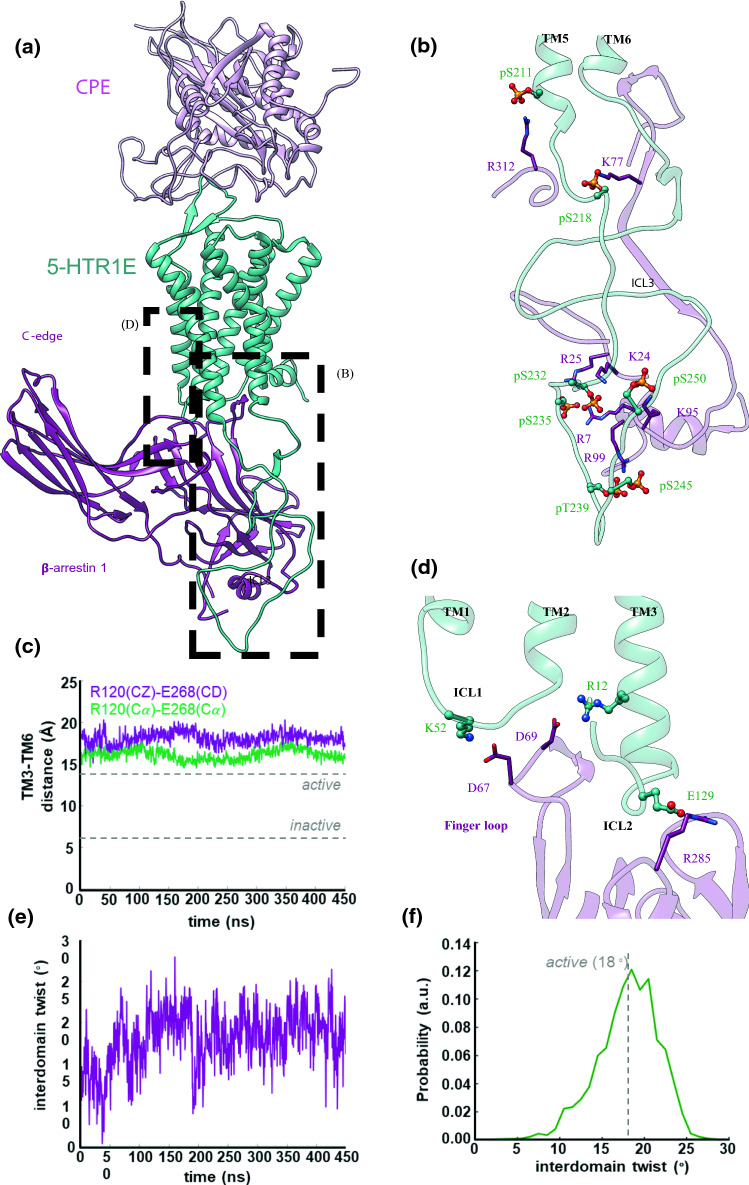
High-affinity CPE–5-HTR1E–β-arrestin1 complex. **a** The side view of the fully active CPE–5-HTR1E–β-arrestin1 complex derived from ~ 1.5 μs MD simulations colored by subunits: CPE (pink), 5-HTR1E (green), and β-arrestin1 (purple). **b** Tight coupling between the phosphorylated ICL3 of 5-HTR1E and the N-domain of β-arrestin1, mostly involving the salt bridges from pS and pT to positively charged residues on the N-domain of β-arrestin1. **c** Analysis of the active-state conformation of 5-HTR1E upon recruiting β-arrestin1. **d** Ionic anchors from β-arrestin1 to the ICL1 and ICL2 of 5-HTR1E. **e** Analysis of the conformation of the β-arrestin1 upon engaging the 7TM core of 5-HTR1E in the presence of CPE. To evaluate the inter-domain twist, we superimposed the N-domain of the active (obtained from our MD simulation) to the inactive (resolved by experiment, PDB ID: 1G4M) state of the β-arrestin1. **f** Distribution of the inter-domain twist angle once the of β-arrestin1 binds the 5-HTR1E

To investigate the cellular signaling mechanism regulated through arrestin pathways, we carried out molecular dynamics studies to predict the interactions of the high-affinity NF-α1/CPE–5-HTR1E complex with β-arrestin 1. An overview of our optimized protein construct is shown in Fig. 5a and suppl. Fig. SK5. Previous studies showed that phosphorylation of intracellular loop (ICL) 3 in GPCRs, plays a crucial role in the recruitment and activation of β-arrestins [35–37].

In order to predict the interaction of β-arrestin 1 with the NF-α1/CPE-5-HTR1E complex, we started with the cryo-EM M2 muscarinic receptor-β-arrestin 1 complex (PDB ID: 6U1N) [38] as a template and superimposed 5-HTR1E on the M2 muscarinic receptor to insert the β-arrestin 1 into the cytoplasmic region of NF-α1/CPE–5-HTR1E complex after phosphorylating the Ser and Thr in the C-terminus and ICL3. Subsequently, we immersed the predicted NF-α1/CPE-5-HTR1E-β-arrestin 1 complex into a lipid bilayer composed of 290 palmitoyl-2-oleoyl-sn-glycero-3-phosphocholine (POPC) molecules. Then we solvated this system with water and ions (sodium chloride) to neutralize the system at the physiological concentration of 0.15 M. This leads to ~ 185,000 atoms in a simulation box of 100 × 100 × 172 Å^3^. We assigned the protonation state of protein residues at the physiological pH = 7.4. We carried out an aggregate of ~ 1.4 μs molecular dynamics simulations to optimize our protein construct. We then used the results of the last 450 ns frames for the analysis presented in this study.

Although the long C-tail and ICL3 are primary sites of phosphorylation in GPCRs [35–37], the long C-tail of 5-HTR1E does not feature any serine or threonine residues to be phosphorylated, leaving the ICL3 with 14 serine and 6 threonine as candidate phosphorylation sites for β-arrestin recruitment. However, the phosphorylation state of these 20 residues is not available from experiment (neither from this study nor from previous works). Thus, in our MD studies, we considered full phosphorylation at all serine (pS) and threonine (pT) residues on the ICL3. This follows the same procedure [39] that we used successfully to study the recruitment of β-arrestin 2 by m-opioid receptor. Our MD studies reported here show that the fully phosphorylated ICL3 interacts strongly with the N-domain of β-arrestin 1, leading to the emergence of numerous salt bridge interactions (Fig. 5b): pS211-R312, pS218-K77, pS235-R25, pS235-R7, pT239-R99, pS245-R99, and pS250-K95, which play a pivotal role in stabilizing the fully activated state of β-arrestin 1 in the NF-α1/CPE-5-HTR1E-β-arrestin 1 complex (Fig. 5a).

Thus, the fully active NF-α1/CPE-5-HTR1E complex facilitates β-arrestin recruitment. We find that the finger loop of β-arrestin 1 penetrates deep into the core of 5-HTR1E to establish a persistent salt bridge from D69 to R120^3.50^ (Fig. 5d). This salt bridge interaction prevents ionic lock formation between R120^3.50^ (CZ)-E268^6.30^(CD), allowing 5-HTR1E to adopt a fully open cytoplasmic region.

We find that the C-edge loops, ^189^QFLMSDKP^196^, ^223^NTNKTVKKI^231^, and ^329^VSRGGLLGDLASS^341^ of the β-arrestin1 anchor extensively to the lipid bilayer, allowing β-arrestin1 to maintain its active conformation with an averaged inter-domain twist angle of ~ 18.8° (Fig. 5e–f). The lipid anchors serve a pivotal role in stabilizing the high-affinity arrestin–GPCR complex since we find that elimination of this lipid-anchoring transits causes the fully active conformation from relax- to inactive-state conformation [38, 39]. Interestingly, our MD simulation indicates that the activated β-arrestin1 couples the phosphorylated 5-HTR1E by forming ionic anchors to ICL1: D67-K52^ICL1^ and ICL2: R285-E129^ICL2^ (Fig. 5d), which sterically discourage Gi protein from coupling to the same binding site in the 5-HTR1E.

### 5-HTR1E and NF-α1/CPE are co-localized in human hippocampal neurons

**Fig. 6:**
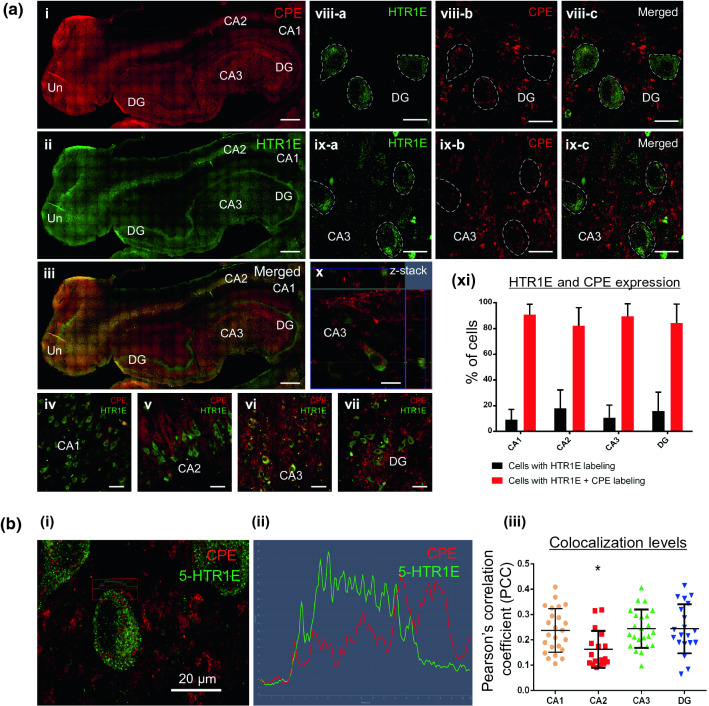
**a** Distribution and cellular co-expression of CPE and 5-HTR1E in human hippocampus. Mosaic of confocal microscope images at 10 × of human hippocampus (Bregma 17.2 mm) showing distribution of CPE (i), 5-HTR1E (ii) and merged (iii). Example of CPE and 5-HTR1E co-expression in neurons in CA1 (iv), CA2 (v), CA3 (vi) and within the hilus in DG (vii). High-magnification images showing co-expression of 5-HTR1E and CPE in CA3 pyramidal neurons (viii-a-c) and DG neurons (ix-a-c). Z-stack of CA3 pyramidal neurons (x) illustrates the distributions and co-expression of CPE and 5-HTR1E along the thickness of the *z*-axis. (xi)The co-expression of CPE and 5-HTR1E was observed in four different human hippocampus and in rostral and caudal sections as well. A total of 190 cells taken from high magnification images as illustrated in **b** were quantified. Bar graphs show the % of cells expressing 5-HTR1E and cells co-expressing 5-HTR1E and NF-α1/CPE in the different hippocampal sub-regions. The %s within the different hippocampal sub-regions are very similar. No significant differences were observed either in 5-HTR1E-expressing cells or in 5-HTR1E- and NF-α1/CPE-co-expressing cells between the CA1, CA2, CA3 and DG (p = 0.4647; one-way ANOVA test). Scale bar: i-iii = 1000 µm, iv-v = 50 µm, vi-vii = 100 µm and viii-x = 20 µm. **b** Co-localization levels of CPE and 5-HTR1E on cell membrane. (i, ii): Image of the part of cell where profile was performed (left). Graph showing the intensity of labeling of both markers across the distance of the profile (right). Scale bar = 20 µm. (iii): Pearson’s correlation coefficient (PCC) of 84 regions of interest (ROI) [CA1 (24 cells), CA2 (17 cells), CA3 (23 cells) and DG (20 cells)] were quantified. Levels of co-localization of the 5-HTR1E and NF-α1/CPE on the cell surface membrane are presented from 0 (no correlation) to 1 (perfect correlation). CA2 shows lower levels of co-localization in contrast to CA1, CA3 and DG sub-regions (*p = 0.0102; one-way ANOVA test). The level of co-localization is consistent with ligand interaction with receptors being a dynamic phenomenon

**Fig. 7:**
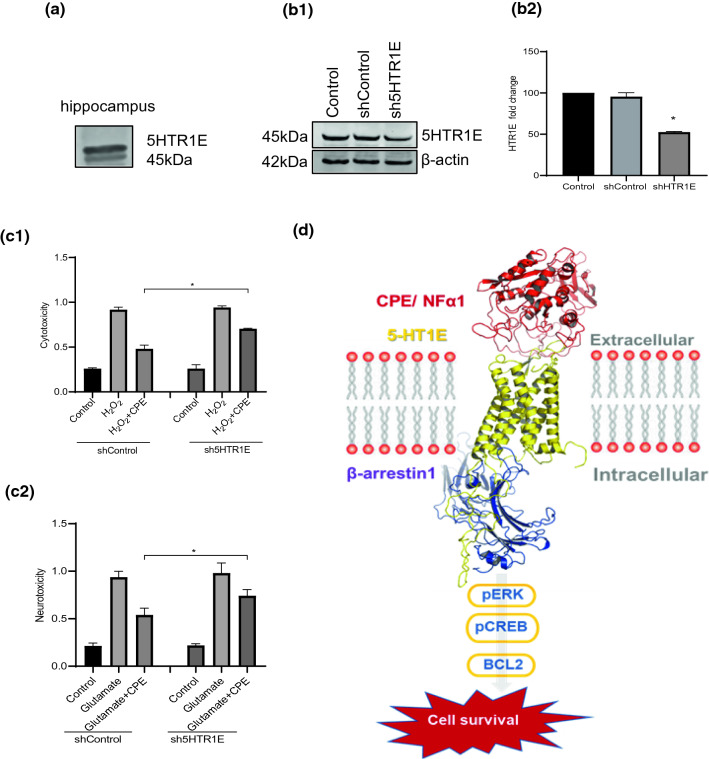
**a** 5-HTR1E expression in human hippocampus. **b** 5-HTR1E–NF-α1/CPE interaction protects human neurons against H_2_O_2_-induced oxidative stress.** b1** Western blot and **b2** image J quantification showing 48% KO of 5-HTR1E in human neurons treated with AV-5-HTR1E shRNA compared to control AV for 72 h. [*F* (2, 3) = 170.0, *p* = 0.0008], *p = 0.0008 for Control vs sh-5-HTR1E, *p* = 0.3266 for Control vs shControl, values are mean ± SD, *n* = 2. **c1, c2** Bar graphs show decreased neuroprotective effect of NF-α1/CPE in 5-HTR1E-KO vs control neurons, against H_2_O_2_- or glutamate-induced cytotoxicity assessed by LDH assay. One-way ANOVA analysis followed by Tukey’s post hoc multiple comparison test. For H_2_O_2_ experiments (**c1**) [*F* (6, 14) = 533.0, *p* < 0.0001] *p < 0.0001 for CPE + H_2_O_2_ in control neurons compared to CPE + H_2_O_2_ in 5-HTR1E-KO neurons. For glutamate experiments (**c2**) [*F* (6, 14) = 118.2, *p* < 0.0001] *p = 0.0170 for CPE + glutamate in control neurons compared to CPE + glutamate in 5-HTR1E- KO neurons. Values are mean ± SD, *N* = 3. **d** Model of Proposed NF-α1/CPE–5-HTR1E signaling mechanism

To determine if 5-HTR1E co-expresses with NF-α1/CPE in the human brain, we carried out immuno-histochemical and immunofluorescence studies on post-mortem human brains (Suppl. Table S5, Fig. 6a, b, Fig. S5–S9). These and Western blot studies (Fig. 7a) revealed that 5-HTR1E and NF-α1/CPE [40] were present in the human hippocampus (Fig. 6ai–iii). Low-magnification images show co-expression of both 5-HTR1E and NF-α1/CPE in neurons in the pyramidal cell layer in Cornu Ammonis areas CA1, CA2 and CA3 and within de Hilus in dentate gyrus (DG) (Fig. 6aiv–vii and Suppl. Fig. S5, S6, S9 using 2 different 5-HTR1E antibodies). Images at higher magnification show 5-HTR1E is located mainly within the perikarya of pyramidal cells and within the granule cell layer of DG. NF-α1/CPE is found in the perikarya of pyramidal cells, the apex, and terminal dendrites along the stratum radiatum (Sr) in CA areas (Fig. 6a viii–x). High-magnification images represented in Fig. 6b were used to quantify the co-expression frequency of NF-α1/CPE. Up to 90% of cells expressing 5-HTR1E co-expressed with NF-α1/CPE in the different sub-regions of the hippocampus. No statistical differences were found when comparing both % of cells co-expressing 5-HTR1E and NF-α1/CPE and % of cells expressing only 5-HTR1E in any of the different hippocampal sub-regions (Fig. 6a xi). Analysis of 5-HTR1E and NF-α1/CPE labeling at the cell membrane suggests that they co-localize and could potentially interact within human hippocampal CA3 neurons (Fig. 6b i–ii), as well as within CA1, CA2 and DG neurons (Suppl. videos 1–4). Z-stack reconstructions and quantitative co-localization analysis were performed to confirm and determine the levels of co-localization between 5-HTR1E and NF-α1/CPE in hippocampal neurons. The analyses showed cell membrane co-localization of 5-HTR1E and NF-α1/CPE at similar levels in the different hippocampal subfields except for CA2 where co-localization levels were lower (Fig. 6b iii). These results suggest possible functional interaction between NF-α1/CPE and 5-HTR1E in human neurons in vivo.

### Down-regulation of 5-HTR1E expression inhibits neuronal cell survival

To determine if 5-HTR1E interaction with NF-α1/CPE could mediate neuroprotection of human primary neurons, we down-regulated 5-HTR1E expression and challenged these neurons with H_2_O_2_ or glutamate to induce oxidative or neurotoxic stress, respectively, in the presence of NF-α1/CPE. Treatment of neurons with adenovirus–5HTR1E–shRNA reduced 5-HTR1E expression by ~ 50% (Fig. 7b1, b2). Figure 7c1 and c2 shows that pre-treatment with NF-α1/CPE effectively reduced H_2_O_2_ or glutamate-induced cyto/neurotoxicity in cultured human primary neurons. However, when these neurons were down-regulated in expression of 5-HTR1E, the effect of pretreatment with NF-α1/CPE on the reduction of cyto/neurotoxicity when challenged with H_2_O_2_ or glutamate was significantly less compared to control neurons_._ Thus, 5-HTR1E plays a pivotal role in protecting human neurons against oxidative and neurotoxic stress-induced cell death, through interaction with NF-α1/CPE.

## Discussion

### Serotonin receptor 5-HTR1E interaction with NF-α1/CPE promotes cell survival

Identifying molecular systems that can protect neurons against various type of stress is important to prevent cognitive dysfunction and neurodegenerative diseases. In this study, we have discovered that the interaction between a new trophin, NF-α1/CPE, and the 5-HTR1E GPCR, a member of the serotonin receptor family with previously unknown function in the brain, produces a strong effect on promoting neuronal survival during oxidative and neurotoxic stress. Interestingly, this cell protective system seems to have evolved for humans and primates since the *HTR1E* gene is not found in mice or rats [19, 41]. Using HEK293 human cells as a model system, we showed that NF-α1/CPE can prevent H_2_O_2_-induced cytotoxicity in 5-HTR1E-transfected HEK293 cells, but not in mock-transfected HEK293 cells.

Most importantly, we showed that 5-HTR1E–NF-α1/CPE interaction protected human primary neurons from cell death when challenged with H_2_O_2_ or glutamate, which induced oxidative or neurotoxic stress, respectively. Knock-down of 5-HTR1E reduced this neuroprotective activity in neurons subjected to oxidative/neurotoxic stress. These findings demonstrate an important role of 5-HTR1E as a receptor for NF-α1/CPE in mediating neuroprotection in human neurons. Since it is known that NF-α1/CPE is highly expressed in stress-vulnerable hippocampal CA3 neurons, in the human brain [40], and is critical in the prevention of stress-induced hippocampal neuronal cell death in mice [4], we carried out immuno-histochemical studies on post-mortem human brains to determine if there is co-expression and co-localization of 5-HTR1E and NF-α1/CPE in hippocampal neurons that could indicate the possibility of functional interaction of these molecules in vivo. Immunofluorescence studies revealed that 5-HTR1E and NF-α1/CPE are co-expressed in the same pyramidal neurons in the CA1, CA2, and CA3 region, and in the dentate gyrus (DG) neurons. This is a broader distribution of 5-HTR1E than in the guinea pig hippocampus where expression was only reported in the DG [22]. Z-stack reconstructions indicate co-localization of NF-α1/CPE and 5-HTR1E at the cell membrane of hippocampal neurons. These data strongly suggest that NF-α1/CPE after secretion from the neuron, could bind to 5-HTR1E to mediate neuroprotection and cell survival in the human hippocampus, in an autocrine/paracrine manner.

### NF-α1/CPE interacts with 5-HTR1E to activate β-arrestin/ERK/BCL2 pathway

To elucidate the mechanism of action of 5-HTR1E, we first demonstrated using luciferase reporter assay [24] that NF-α1/CPE was able to activate 5-HTR1E in HTLA cells after treatment with recombinant NF-α1/CPE extracellularly. Our radioligand-binding experiments showed that NF-α1/CPE binds to 5-HTR1E specifically and in a saturable manner with high affinity, (Kd = 13.82 nM) when 5-HTR1E-expressing HEK293 stable cells were treated with [^125^I]NF-α1/CPE. The binding affinity of NF-α1/CPE to 5-HTR1E was lower than some, and similar to other glycoprotein hormones binding to their respective GPCRs [42–44]. Interaction between NF-α1/CPE and 5-HTR1E was further confirmed in LN-18 cells, a human glioblastoma cell line that expresses both the ligand and receptor endogenously. Co-immunoprecipitation assays showed that NF-α1/CPE antibodies pulled down 5-HTR1E, indicating interaction between these two molecules. Furthermore, our pull-down experiments showed that full-length NF-α1/CPE and a CPE fragment comprising residues 1–350, but not fragments 1–150 or 351–476 interacted with 5-HTR1E. This finding indicated that the binding domain of NF-α1/CPE to 5-HTR1E is likely within residues 151–350 which corroborated with our molecular dynamics studies. It is of note that there is always a possibility that improper folding could affect results of pull-down assays. Hence, future testing of the binding activities between CPE and 5-HTR1E after mutating specific amino acid residues on CPE predicted to interact with 5-HTR1E by molecular docking and dynamics studies, will be carried out to further confirm our model experimentally.

Our studies showed that NF-α1/CPE-5-HTR1E activated a signal transduction mechanism leading to cell survival which involved ERK phosphorylation. Upon NF-α1/CPE binding to 5-HTR1E stable cells, there was a 3.4-fold increase in pERK1/2 compared to normal HEK293 cells. It has been reported that ERK phosphorylation leads to activation of its downstream protein CREB, which then regulate the level of the pro-survival protein BCL2 to protect cells against oxidative stress [45, 46]. Our studies found that NF-α1/CPE increased phosphorylation of CREB up to 30% in 5-HTR1E-expressing cells compared to controls. Furthermore, after treatment with H_2_O_2_, to induce oxidative stress, the decrease in BCL2 protein level was prevented in NF-α1/CPE-treated 5-HTR1E stable cells as compared to normal HEK293 cells. Thus, NF-α1/CPE-5–HTR1E interaction promotes cell survival via activation of the ERK/CREB/BCL2 pathway.

GPCRs can stimulate ERK phosphorylation through different pathways, such as through G proteins [47] or through recruitment of arrestin which can function as an independent mediator of G proteins [48]. Since 5-HTR1E is a Gi-linked receptor [17], we tested the Gi inhibitor, PTX, and a Gq inhibitor FR900359 [34], and found that neither inhibitor had an effect on inhibiting NF-α1/CPE activation of ERK expression in 5-HTR1E stable cells. However, we were able to show that the NF-α1/CPE-induced ERK 1/2 phosphorylation in 5-HTR1E stable cells was diminished in HEK293–β-arrestin–KO cells. This finding indicates that β-arrestin is critical in HTR1E-mediated ERK phosphorylation. Furthermore, upon treatment with NF-α1/CPE, 5-HTR1E-expressing β-arrestin KO HEK293 cells were unable to mitigate the H_2_O_2_-induced cytotoxicity compared to control 5-HTR1E- expressing HEK293 cells. Thus, arrestin recruitment by the NF-α1/CPE–5-HTR1E complex is pivotal in activating the ERK/CREB/BCL2 pathway.

It is known that 5-HTR1E, upon binding with 5HT inhibits the cAMP pathway [17, 49]. However, when we treated 5-HTR1E stable cells with NF-α1/CPE, 5-HT or BRL54443 (5-HTR1E agonist) in the presence of forskolin, we found no effect of NF-α1/CPE on forskolin-stimulated cAMP, while 5-HT or BRL54443 both reduced cAMP levels significantly. This result shows that the NF-α1/CPE–5-HTR1E complex does not activate cAMP signaling which is limited to 5-HTR1E stimulation by serotonin. While serotonin was able to induce ERK signaling in 5-HTR1E stable cells, it did not up-regulate BCL2 protein expression (Fig. S4), suggesting that the neuroprotection effect resulting from interaction of NF-α1/CPE with 5-HTR1E is independent of serotonin interaction with this receptor.

### Structural mechanism for NF-α1/CPE and 5-HTR1E interaction and recruitment of β-arrestin, independent of serotonin pocket

Molecular dynamics (MD) studies were carried out to understand the interactions at the molecular level between NF-α1/CPE and 5-HTR1E, and subsequent recruitment of β-arrestin. First, we generated an active structure for 5-HTR1E and predicted that the binding of CPE to 5-HTR1E, is not at the serotonin-binding pocket. Instead, we found that NF-α1/CPE interacts with the ECL1/ECL2 of 5-HTR1E via 3 stable salt bridges composed of K302-D86^ECL1^, D306-K89^ECL1^, D275-R165^ECL2^. Then using MD techniques to phosphorylate the Ser and Thr in ICL3 of 5-HTR1E and allowing it to interact with β-arrestin1, we found very strong coupling of β-arrestin1 to ICL2 and ICL3 of 5-HTR1E, leading to activation of β-arrestin1. This demonstrates how the binding of NF-α1/CPE to 5-HTR1E can induce β-arrestin1 activation, which we conclude is responsible for the cell survival effect provided by NF-α1/CPE interacting with 5-HTR1E.

## Conclusion

We have discovered a new role for 5-HTR1E serotonin receptor, which previously had no neurophysiological function. The novel interaction of 5-HTR1E with NF-α1/CPE uncovered a new mechanism for promoting neuroprotection of human neurons during induced oxidative and neuroexcitotoxic stress (Fig. 7d). Cell biological and molecular dynamics studies indicate that the mechanism of signaling for 5-HTR1E to promote cell survival involves activation of the ERK–CREB–BCL2 signaling pathway, via recruitment of β-arrestin, which is infrequent among GPCRs. Since the *5-HTR1E* gene is expressed in humans and primates, but not rats or mice, this cell survival/protective mechanism may be of specific importance to *Homo sapiens,* especially in the human hippocampus where both molecules are poised for interaction since they are co-localized in neuronal surface membranes. Interestingly, since our previous work on mice and rats suggests a similar receptor-mediated mechanism in the transduction of neuroprotective activity of NF-α1/CPE [4, 13], other receptors must be involved and yet to be identified in these species. Our discovery and molecular understanding of how 5-HTR1E interacts with NF-α1/CPE to promote neuronal survival pave the way for design of small molecule agonists to the 5-HTR1E for potential therapeutic use in preventing and treatment of human neurodegenerative diseases.

## Supplementary Information

Below is the link to the electronic supplementary material.

Supplementary file1 (DOCX 43557 KB)

Supplementary file2 (MP4 830 KB)

Supplementary file3 (MP4 4481 KB)

Supplementary file4 (MP4 1905 KB)

Supplementary file5 (MP4 1063 KB)

## Data Availability

The data sets generated during and/or analyzed during the current study are available from the corresponding author on reasonable request.
