# Supplementary material for: Novel interaction between neurotrophic factor-α1/carboxypeptidase E and serotonin receptor, 5-HTR1E, protects human neurons against oxidative/neuroexcitotoxic stress via β-arrestin/ERK signaling
Source: Cell Mol Life Sci. 2021 Dec 29;79(1):24. doi: 10.1007/s00018-021-04021-3 (PMC8732845; doi:10.1007/s00018-021-04021-3)
Supplement: Supplementary file 1 — Supplementary file1 (DOCX 43557 KB) [file 18_2021_4021_MOESM1_ESM.docx]

**SUPPLEMENTARY INFORMATION FOR**

**Novel interaction between Neurotrophic factor-α1/Carboxypeptidase E and serotonin receptor, 5-HTR1E, protects human neurons against oxidative/neuroexcitotoxic stress *via* β-arrestin/ERK signaling**

Vinay Kumar Sharma^1^, Xuyu Yang^1^, Soo-Kyung Kim^2#^, Amirhossein Mafi^2#^, Daniel Saiz-Sanchez^3#^, Patricia Villanueva-Anguita^3^, Lan Xiao^1^, Asuka Inoue^4^, William A.Goddard III^2*^ and Y. Peng Loh^1*^

^1^Section on Cellular Neurobiology, *Eunice Kennedy Shriver* National Institute of Child Health and Human Development, National Institutes of Health, Bethesda, MD 20892, USA

^2^Materials and Process Simulation Center, California Institute of Technology. Pasedena, CA 91125, USA

^3^Neuroplasticity and Neurodegeneration Laboratory, Medical School, Regional Center for Biomedical Research, University of Castilla-La Mancha, Ciudad Real 13071, Spain

^4^Graduate School of Pharmaceutical Sciences, Tohoku University, Miyagi, 980-8578 Japan

# these three authors contributed equally

**Short title**: NFα1-5-HTR1E interaction mediates cell survival

**Key words**: GPCR, β-arrestin, cytotoxicity, neuroprotection

**Co-corresponding authors:**

Dr. Y. Peng Loh,

Section on Cellular Neurobiology,

49, Convent Drive, Bldg 49, Rm 6A-10,

NICHD, NIH, Bethesda, Md. 20892, USA.

Email: [lohp@mail.nih.gov](mailto:lohp@mail.nih.gov); Tel. (301)4963239

and

Prof. William A. Goddard III

Materials and Process Simulation Center,

California Institute of Technology, Pasadena, CA. 11125, USA.

E-mail: [wag@caltech.edu](mailto:wag@caltech.edu) and wagoddard3@gmail.com

**Supplementary methods**

**Supplementary method S1. Molecular docking of NFα1/CPE with 5-HTR1E**

In order to predict which NF-α1/CPE residues bind to 5-HTR1E surface residues, both NF-α1/CPE and 5-HTR1E structures were predicted using available templates in the PDB database. We then used these structures to predict the binding site for CPE.

**Active 5-HTR1E generation.** In order to predict active human 5-HTR1E, we predicted the ensemble of low energy G protein coupled receptors (GPCR) structures using the GPCR Ensemble of Structures in Membrane BiLayer Environment (GEnSeMBLE) Monte Carlo method [1-4]. GEnSeMBLE identifies the TM domains based on multiple sequence alignments, then selects the shape of each helix based on the force field or on homology to known structures, and then considers a complete set (~13 trillion combinations) of rigid rotations and tilting of the helices, to select ~25 that are sufficiently stable to play a role in binding of ligands and activation. For each of the 12 interacting pairs of helices, we optimize side chain conformations using SCREAM (Side Chain Rotamer Excitation Analysis Method) [5] followed by 10 steps of conjugate gradient minimization to remove bad contacts. These pairwise energies are then used to evaluate the energies of all 13 trillion combinations, from which the 2,000 lowest energy combinations are selected for the next step. All simulations used the Dreiding force field [6]. Since the X-ray structures of 7 5-HT serotonin receptors (four 5-HT1B, 2 5-HT2C, 1 5-HT2B) were available (Table S1), we used the 5-HT crystal structure itself as the template to define the rigid shape to be rotated. Among 7 experimental structures of 5-HT serotonin receptors, 5 X-ray templates (5-HT1B with dihydroergotamine or ergotamine agonist, 5-HT1B with donitriptan agonist and Go protein, 5-HT2C with ergotamine agonist, and 5-HT2B with ergotamine agonist) are available for active state of TM helix. For the current application, we used 4 templates, 5-HT1B with ergotamine agonist (PDB: 4IAR), [7] 5-HT1B with donitriptan agonist and Go protein (PDB: 6G79), [8] 5-HT2C with ergotamine agonist (PDB: 6BQG), [9] and 5-HT2B with ergotamine agonist (PDB: 5TUD) [10].

**Table S1**. The currently available experimental structures of 5-HT serotonin receptors.

| # | UnitProtID | PDB | Subtype | Ligand | Resolution (Å) |
| --- | --- | --- | --- | --- | --- |
| 1 | P28222 | 4IAQ | 5-HT1B | Dihydroergotamine agonist | 2.80 |
| 2 | P28222 | 4IAR | 5-HT1B | Ergotamine agonist | 2.70 |
| 3 | P28222 | 5V54 | 5-HT1B | Methiothepin inverse agonist | 3.90 |
| 4 | P28222 | 6G79 | 5-HT1B | Donitriptan agonist, Go protein | 3.78 |
| 5 | P28335 | 6BQG | 5-HT2C | Ergotamine agonist | 3.00 |
| 6 | P28335 | 6BQH | 5-HT2C | Ritanserin inverse agonist | 2.70 |
| 7 | P41595 | 5TUD | 5-HT2B | Ergotamine agonist | 3.00 |

Next we added the loops of the cryo-electron microscopy (cryo-EM) structure of active 5-HT1B (PDB: 6G79)[8] back to the final active structure from GEnSeMBLE; N-term: 18,19, IC1: 50-55, EC1: 85-91, IC2: 126-135, EC2: 161-177 (161-170 missing), IC3: 212-283 (215-281 missing), EC3: 315-320 (315-319 missing), H8: 348-360. The disulfide bond was constructed between C95 and C173. After 10 cycles of simulated annealing from 50 to 600 K with fixed the secondary structures, the lowest energy structure was used for docking to NFα1/CPE.

**Full solvent MD for mouse NF-α1/CPE predicted model**. The waters and ions were added using VMD. Ions were added to neutralize the system, and then added up to physiological conditions. The unit cell size for the entire protein was then 80 ˚A by 80 ˚A by 110 ˚A.

With the protein fixed, the solvent and membrane were first minimized using NAMD and the forcefield CHARMM-22. Dynamics on just the solvent were then performed for 0.5 ns with 1 fs time steps. The temperature of the system was implemented with Langevin dynamics with damping with a bath temperature of 310 K. Pressure monitoring was also performed with NAMD’s Langevin piston at 1 atm.

After solvent and membrane equilibration, the protein were unfixed, and the structure minimized again. Full dynamics of the system were then run for 5 ns with the helix constraints and then another 5ns without any constraints. We then check the lowest energy structure during the trajectories.

**Full solvent full membrane MD for 5-HTR1E and NF-α1/CPE complex.** We prepositioned NF-α1/CPE to make 4 salt-bridges with 5-HTR1E between 1) K89 in EC1 of 5-HTR1E and E268 in NFα1/CPE, 2) R161 in EC2 of 5-HTR1E and D306 in NFα1/CPE, 3) R164 in EC2 of 5-HTR1E and D305 in NFα1/CPE, and 4) R165 in EC2 of 5-HTR1E and D304 in NF-α1/CPE, following by minimization with salt bridge constraints.

The lipid membrane, waters and ions were added using VMD. Ions were added to neutralize the system, and then added up to physiological conditions. The unit cell size for the entire protein was then 85 ˚A by 85 ˚A by 75 ˚A.

With the protein fixed, the solvent and membrane were first minimized using NAMD and the forcefield CHARMM-22. Dynamics on just the membrane and solvent were then performed for 0.5 ns with 1 fs time steps. The temperature of the system was implemented with Langevin dynamics with damping with a bath temperature of 310 K. Pressure monitoring was also performed with NAMD’s Langevin piston at 1 atm.

After solvent and membrane equilibration, the protein were unfixed, and the structure minimized again. Full dynamics of the system were then run for 100 ns with the same parameters as in the solvent equilibration including 5 ns with 4 salt bridge constraints. We then check to determining if all the contacts found in the docking are maintained.

**Supplementary method S2. Molecular dynamics simulation of β-arrestin with 5-HTR1E** To form the fully active complex of CPE-5-HTR1E-β-arrestin1, we used the cryo-EM structure of M2 muscarinic- β-arrestin1 (PDB ID:6U1N)[11] as a template. We superimposed the 5-HTR1E onto the M2 muscarinic receptor and inserted the β-arrestin1 into the 5-HTR1E-CPE complex we obtained from our molecular docking calculations (see SI appendix). In addition, we optimized the long ICL3 loop using the MODELLER program [12], and then we phosphorylated all Ser and Thr residues on ICL3 and the C terminus loop of 5-HTR1E. We subjected the resulting complex of CPE-5-HTR1E- β-arrestin1 to 1000 steps of energy minimization using the steepest descents algorithm and then performed 4000 cycles of simulated annealing simulation over a total of 200 ns, where all residues were heated from 0 to 600K over 20 ps and then cooled back to 310K over 30 ps. During this process, all backbone atoms except the residues on ICL3, were restrained with a force constant of 9.6 kcal.mol^-1^.Å^-2^, This allowed ICL3 to wrap around the β-arrestin1 to establish strong interactions. We immersed the refined complex of CPE-5-HTR1E- β-arrestin1 into the lipid membrane (290 POPC molecules), water, and ions to neutralize the system at the physiological concentration of 150 mM. This leads to ~185,000 atoms in a simulation box of 100×100×172 Å^3^. We assigned the protonation state of the protein residues at the physiological pH=7.4. We carried out a ~300ns MD simulation in which positional restraints were placed on the heavy atoms with a force constant of 9.6 kcal.mol^-1^ Å^-2^. In addition, we restrained the z-coordinate of the POPC headgroups inside the membrane with a force constant of ~2.4 kcal.mol^-1^Å^-2^ to allow the POPC molecules to move freely within the xy-plane to find their appropriate packing. Throughout this calculation, the restraints on the protein, and POPC were gradually reduced to 0 kcal.mol^-1^Å^-2^, which prepared the construct for the further relaxation. Subsequently, we performed a ~900ns MD simulation with GROMACS [13] without constraints to optimize and equilibrate the protein complex. We used the last ~450ns trajectory for making Figures 2 and Figure 5.

**Force fields and algorithms.**

In our full-atomistic simulations, all molecules: protein, POPC and ions were described using the Charmm36m [14] parameter set. Water was described using the TIP3P model[15]. For all calculations, the temperature was maintained at 310K using a velocity-rescale[16] thermostat with a damping constant of 1.0 ps for temperature coupling while the pressure was controlled at 1 bar using a Parrinello-Rahman barostat algorithm [17] with a 5.0 ps damping constant for the pressure coupling. Semi-isotropic pressure coupling was used during this calculation. The Lennard-Jones cutoff radius was 12 Å, where the interaction was shifted smoothly to 0 after 10 Å. Periodic boundary conditions were applied to all three directions. We used the Particle Mesh Ewald algorithm [18] with a real cutoff radius of 12 Å and a grid spacing of 1.2 Å to calculate the long-range coulombic interactions. We used a compressibility of 4.5 ×10^-5^ bar^-1^ in the xy- plane and along the z axis, to relax the box volume. In all the above simulations**,** water OH-bonds were constrained by the SETTLE algorithm [19]. The remaining H-bonds were constrained using the P-LINCS algorithm [20].

**Supplementary method S3. Immunofluorescence and immunohistochemistry of human hippocampus.**

Immunofluorescence and immunohistochemistry experiments were performed in six postmortem brain tissues (without neuropathology) which were obtained from three national brain banks (Barcelona IDIBAPS, Murcia BIOBANC-MUR and Madrid BTCIEN, Spain). All the experimental procedures were approved by the Ethical Committee of Clinical Research at Ciudad Real University Hospital (grant numbers SAF2016-75768-R and SBPLY/17/180501/000430). Six cases without neuropathology were used for Immunofluorescence and immunohistochemistry (Suppl. Table 5). Cases BCM-587, BCPA-449, BCM-143 and BCM-104 were used for double immunofluorescence, case BCM-98 was used for immunohistochemistry. For immunohistochemistry and immunofluorescence, all tissue blocks were post-fixed in fresh phosphate-buffered 4% paraformaldehyde for 45 days upon receipt to standardize the conditions of the samples. Tissue blocks were then immersed in a phosphate-buffered solution of 2% dimethyl sulfoxide (DMSO) and 10% glycerol for 48 h, followed by immersion in a phosphate-buffered solution of 2% DMSO and 20% glycerol for 48 h. Blocks were cryoprotected with sucrose 30% and coronally sectioned with a freezing sliding microtome (Microm HM 450) in sections of 50 μm thickness. For western blot, all blocks were received and stored at -80ºC. All cases were provided from the three main national brain banks (Barcelona IDIBAPS, Murcia BIOBANC-MUR and Madrid BTCIEN, Spain) and all the experimental procedures were approved by the Ethical Committee of Clinical Research at Ciudad Real University Hospital (grant numbers SAF2016-75768-R and SBPLY/17/180501/000430).

For immunohistochemistry and immunofluorescence tissue antigenicity was unmasked by boiling the tissue under pressure for 2 min in citrate buffer. Sections were immersed in formic acid for 3 min and rinsed in phosphate buffer (PBS 0.01M, pH = 7.4, 3 x 10 min). Human tissue auto fluorescence was reduced by incubation under UV light for 24h (only for immunofluorescence). Endogenous peroxidase activity was inhibited by a 30-min bath in 1% H_2_O_2_ in phosphate-buffered saline. Blocking consisted of 5% NDS + 0.3% Triton X-100 in PBS. Sections were incubated overnight at 4 °C with primary (1:50, rabbit anti-Carboxypeptidase E, Proteintech® (Cat No.13710-1-AP) and/or 1:100, mouse anti-5-HTR1E, Abnova© (Cat. No. H00003354-M03) for Fig S4-S6, or 1:100, rabbit S31 anti-5-HTR1E, Abcam (Cat no. ab236651) for Fig.S7) containing 0.3% Triton X-100 and 5% normal serum in phosphate-buffered saline. Controls included omitting primary antibodies. After washing in PBS (3 x 10 min), sections were incubated for 2 h in secondary biotinylated antibodies (1:200, anti-mouse or 1:200, anti-rabbit, Vector Laboratories, CA, USA) or fluorescent antibodies (1:200, Alexa 488 donkey anti-mouse, Alexa 568 donkey anti-rabbit, Molecular Probes, CA, USA). For immunohistochemistry, sections were then incubated for 2h in avidin-biotin complex (VECTASTAIN® Elite ABC kit) and subsequently washed in PBS (3 × 10 min). Antibodies were revealed with 3,3′-diaminobenzidine (DAB, Sigma-Aldrich) and 0.1% H2O2. Sections were counterstained with cresyl violet and mounted using DPX (BDH, Poole, UK). For immunofluorescence, sections were mounted and coverslipped with antifading polyvinyl alcohol mounting medium (PVA-DABCO®, Sigma-Aldrich, Co). To carry out quantification of both % of cells-expressing 5-HTR1E and cells-expressing 5-HTR1E and NF-α1/CPE, 8 z-stack series were taken from CA1, CA3 and DG and 6 z-stack series were taken from CA2. All cells present within the z-stacks were quantified (N=190). These same z-stacks were used to analyze co-localization levels of 5-HTR1E and NF-α1/CPE. For this, two or three (whenever possible) regions of interest (ROI) corresponding to individual cells presenting membrane staining for 5-HTR1E and NFα1/CPE were selected from each z-stack (N=84). Then, Pearson's Correlation Coefficient (PCC) of both channels (green and red) related to each marker were measured using the specific plugging EzColocalization with Image-J free software [21]. Briefly, this coefficient analyzes the pixel spatial correlation and offers a grade of results from -1 (anti-correlation), 0 (no correlation) to +1 (perfect correlation). Statistical analyses for these data were obtained using one way-ANOVA test. Images of immunohistochemistry were taken with Zeiss Axio Imager M2 microscope and fluorescent images were acquired with Zeiss LSM 800 with Airyscann confocal microscope. All analyses were carried out from Bregma interval 17.2 – 22.6 mm [22] . Controls omitting primary antibodies for NFα1/CPE and 5-HTR1E were carried out. Additional antibody absorption staining was performed to confirm the specificity of mouse anti-HTR1E, Abnova© antibody. Anti-HTR1E was incubated overnight in the presence of antigen HTR1E (0.5mg/mL, Prestige Antigens^™^) and then immunohistochemistry protocol was carried out.

**Supplementary results**

**Supplementary result SK1. Predictions of the active structural ensemble for 5-HTR1E**

Among 12 subtypes of serotonin receptors, 5-HTR1F has the highest sequence identity of 56. 44% in all sequences and 73.77% in TMD with 5-HTR1E (Table S2). In all experimental structures of 5-HTRs in Table 1, 3 subtypes in bold in Table 2 are available for TM helix generation. 5-HT1B has higher sequence identity of 65.28 % in TM than 5-HT2C (44.26%) and 5-HT2B (39.63%) TMD.

**Table S2**. Sequence identities (%) of 5-HTR1E with all subtypes of 5-HT serotonin receptors in all and transmembrane domains (TM) sequences. Three subtypes in bold are available for the experimental structures.

| **#** | **Subtypes** | **All** | **TMs** | **TM1** | **TM2** | **TM3** | **TM4** | **TM5** | **TM6** | **TM7** |
| --- | --- | --- | --- | --- | --- | --- | --- | --- | --- | --- |
| 1 | 5-HTR1E | 100.00 | 100.00 | 100.00 | 100.00 | 100.00 | 100.00 | 100.00 | 100.00 | 100.00 |
| 2 | 5-HT1A | 38.90 | 52.91 | 23.81 | 62.50 | 52.38 | 38.89 | 78.26 | 61.90 | 52.63 |
| 3 | **5-HT1B** | 46.58 | 65.28 | 47.62 | 70.83 | 76.19 | 61.11 | 60.87 | 66.67 | 73.68 |
| 4 | 5-HT1D | 45.48 | 66.69 | 38.10 | 70.83 | 76.19 | 72.22 | 73.91 | 61.90 | 73.68 |
| 5 | 5-HT1F | 56.44 | 73.77 | 38.10 | 87.50 | 76.19 | 77.78 | 86.96 | 76.19 | 73.68 |
| 6 | 5-HT2A | 27.67 | 41.85 | 38.10 | 45.83 | 47.62 | 55.56 | 30.43 | 33.33 | 42.11 |
| 7 | **5-HT2B** | 25.21 | 39.63 | 23.81 | 50.00 | 47.62 | 44.44 | 26.09 | 38.10 | 47.37 |
| 8 | **5-HT2C** | 27.67 | 44.26 | 38.10 | 54.17 | 52.38 | 44.44 | 34.78 | 33.33 | 52.63 |
| 9 | 5-HT4 | 29.32 | 45.32 | 28.57 | 58.33 | 47.62 | 38.89 | 47.83 | 38.10 | 57.89 |
| 10 | 5-HT5A | 29.59 | 45.36 | 23.81 | 58.33 | 38.10 | 44.44 | 47.83 | 52.38 | 52.63 |
| 11 | 5-HT6 | 25.75 | 38.49 | 28.57 | 33.33 | 47.62 | 27.78 | 26.09 | 42.86 | 63.16 |
| 12 | 5-HT7 | 33.15 | 52.48 | 33.33 | 54.17 | 52.38 | 61.11 | 60.87 | 47.62 | 57.89 |

The starting templates for GEnSeMBLE predictions were the crystal structures of 4 active templates bound with agonists, 1) 5-HT1B (PDB: 4IAR),[7] 2) 5-HT1B with Go protein (PDB: 6G79),[8] 3) 5-HT2C (PDB: 6BQG),[9] and 4) 5-HT2B (PDB: 5TUD).[10] To find the optimum packings of the 7-helix bundle, we applied the BiHelix and ComBiHelix on the TM region [1]. This process sampled the η-angles from 0° to 360° in 30° increments to generate (12)^7^=35 million combinations. The top 10 helix orientations are shown in Table 3. HT1B templates shows much lower E than HT2B (#659) and HT2C (#212). All 0 structure of HT1B (PDB: 4IAR) ranks as top 1 and another all 0 structure of HT1B (PDB: 6G79) rank as top 4, respectively.

**Table S3**. Top10 from BiHeix and ComBihelix ranked by CNTi, the average rank of charged inter-helical (CInt), charged total (CTot), neutral inter-helical (NInt) and neutral total (NTot) energy (kcal/mol). All 0 structures in grey were repeated twice because the program includes the starting structure.

| **#** | **H1** | **H2** | **H3** | **H4** | **H5** | **H6** | **H7** | **PDB_subtype** | **CInt** | **CTot** | **NInt** | **NTot** |
| --- | --- | --- | --- | --- | --- | --- | --- | --- | --- | --- | --- | --- |
| 1 | 0 | 0 | 0 | 0 | 0 | 0 | 0 | 4IAR_HT1B | -503.49 | 201.24 | -464.51 | 96.95 |
| 2 | 0 | 0 | 0 | 0 | 0 | 0 | 0 | 4IAR_HT1B | -503.49 | 201.24 | -464.51 | 96.95 |
| 3 | 0 | 0 | 0 | 15 | 0 | 0 | 0 | 6G79_HT1B | -531.96 | 190.88 | -446.31 | 126.51 |
| 4 | 0 | 0 | 0 | 0 | 0 | 0 | 0 | 6G79_HT1B | -541.01 | 240.37 | -461.03 | 144.16 |
| 5 | 0 | 0 | 0 | 0 | 0 | 0 | 0 | 6G79_HT1B | -541.01 | 240.37 | -461.03 | 144.16 |
| 6 | 0 | 0 | 0 | -15 | 0 | 0 | 0 | 6G79_HT1B | -523.56 | 249.31 | -461.80 | 140.74 |
| 7 | 0 | 0 | 0 | 0 | 15 | 0 | 0 | 6G79_HT1B | -507.64 | 242.21 | -439.80 | 150.38 |
| 8 | 0 | 0 | 0 | 0 | 15 | 0 | 0 | 4IAR_HT1B | -481.30 | 210.70 | -437.39 | 114.08 |
| 9 | 0 | 0 | 0 | -15 | 0 | 0 | 0 | 4IAR_HT1B | -485.73 | 241.61 | -442.80 | 136.62 |
| 10 | 0 | 0 | 0 | 15 | 0 | 0 | 0 | 4IAR_HT1B | -478.08 | 177.83 | -423.69 | 98.68 |

We then selected 2 of these structures based on their energy (top1) and numbers of active contacts (top 4) which has the highest active contacts of 8 and applied the SuperBiHelix and SuperComBiHelix methods[2, 4]. Here we sampled η- and φ-angles from −30° to 30° in 15° increments and −10°, 0° and 10° for the tilt angle, θ, leading to a total of (5*5*3)7=13 trillion combinations, for each of which the energy was evaluated using the SuperBiHelix method. Finally we selected the best 25 structures in Table 4. Most of structures (24 out of 25) are from HT1B (PDB: 4IAR) templates which has better resolution (2.70 Å) compared with HT1B (PDB: 6g79) templates (3.78 Å). However, the structure of HT1B (PDB: 6G79) bound with Go protein has the most active contacts, 7. Thus, top 9 structure from SuperComBihelix in grey was selected to generate the active structure.

**Table S4**. Top25 from SuperBiHeix and SuperComBihelix with the numbers of active (Act), inactive (Inact) and Class A contacts ranked by CNTi, the average rank of charged inter-helical, charged total, neutral inter-helical and neutral total energy.

| **#** | **PDB: 7 θ x 7 φ x** **7 η angles** | **Act** | **Inact** | **ClassA** |
| --- | --- | --- | --- | --- |
| 1 | 4IAR: 0_0_0_0_0_0_0x0_0_0_15_0_-15_0x0_0_0_0_0_0_0 | 3 | 1 | 40 |
| 2 | 4IAR: 0_0_0_0_0_0_0x0_0_0_30_-15_-15_0x0_0_0_0_0_0_0 | 3 | 1 | 39 |
| 3 | 4IAR: 0_0_0_0_0_0_0x0_0_0_30_-15_15_0x0_0_0_0_0_0_0 | 2 | 4 | 38 |
| 4 | 4IAR: 0_0_0_0_0_0_0x0_0_0_30_0_-15_-15x0_0_0_0_0_0_0 | 2 | 2 | 39 |
| 5 | 4IAR: 0_0_0_0_0_0_0x0_0_0_15_-15_15_0x0_0_0_0_0_0_0 | 2 | 4 | 38 |
| 6 | 4IAR: 0_0_0_0_0_0_0x0_0_0_15_0_-15_-15x0_0_0_0_0_0_0 | 1 | 3 | 40 |
| 7 | 4IAR: 0_0_0_0_0_0_0x0_0_0_30_0_-15_0x0_0_0_0_0_0_0 | 3 | 1 | 40 |
| 8 | 4IAR: 0_0_0_0_0_0_0x-15_0_0_30_-15_-30_15x0_0_0_0_0_0_0 | 3 | 3 | 35 |
| 9 | 6G79: 0_0_0_0_0_0_0x0_0_0_-15_0_-15_0x0_0_0_0_0_0_0 | 7 | 0 | 38 |
| 10 | 4IAR: 0_0_0_0_0_0_0x0_0_0_30_0_15_0x0_0_0_0_-15_0_0 | 1 | 4 | 39 |
| 11 | 4IAR: 0_0_0_0_0_0_0x0_0_0_15_-15_-15_0x0_0_0_0_0_0_0 | 3 | 1 | 39 |
| 12 | 4IAR: 0_0_0_0_0_0_0x0_0_0_30_-15_0_0x0_0_0_0_0_0_0 | 3 | 3 | 39 |
| 13 | 4IAR: 0_0_0_0_0_0_0x0_0_0_30_-15_-30_15x0_0_0_0_0_0_0 | 2 | 3 | 39 |
| 14 | 4IAR: 0_0_0_0_0_0_0x0_0_0_-15_0_-15_-15x0_0_0_0_0_0_0 | 1 | 3 | 40 |
| 15 | 4IAR: 0_0_0_0_0_0_0x0_0_0_-15_0_-15_0x0_0_0_0_0_0_0 | 4 | 1 | 39 |
| 16 | 4IAR: 0_0_0_0_0_0_0x0_0_0_30_0_-30_15x0_0_0_0_0_0_0 | 5 | 3 | 40 |
| 17 | 4IAR: 0_0_0_0_0_0_0x0_0_0_-15_-15_15_0x0_0_0_0_0_0_0 | 2 | 4 | 38 |
| 18 | 4IAR: 0_0_0_0_0_0_0x0_0_0_30_0_0_-15x0_0_0_0_0_0_0 | 2 | 3 | 39 |
| 19 | 4IAR: 0_0_0_0_0_0_0x0_0_0_15_0_-30_0x0_0_0_0_0_0_0 | 2 | 2 | 40 |
| 20 | 4IAR: 0_0_0_0_0_0_0x-15_0_0_15_0_-15_0x0_0_0_0_0_0_0 | 2 | 3 | 39 |
| 21 | 4IAR: 0_0_0_0_0_0_0x0_0_0_30_0_-30_0x0_0_0_0_0_0_0 | 2 | 2 | 40 |
| 22 | 4IAR: 0_0_0_0_0_0_0x0_0_0_30_-15_15_0x0_0_0_0_-15_0_0 | 2 | 3 | 39 |
| 23 | 4IAR: 0_0_0_0_0_0_0x0_0_0_0_-15_-15_0x0_0_0_0_0_0_0 | 3 | 1 | 39 |
| 24 | 4IAR: 0_0_0_10_0_0_0x0_0_0_-15_0_-30_0x0_0_0_0_0_0_0 | 2 | 3 | 39 |
| 25 | 4IAR: 0_0_0_0_0_0_0x0_0_0_0_-15_15_0x0_0_0_0_0_0_0 | 2 | 4 | 38 |

Inspection of the three rotational angles shows that the sweep angles φ presents the most variation, especially in TM4, TM5, and TM6. In nearly all structures, TMs differ by 15º or 30º compared with the X-ray structure. These helices are more flexible than others, and may play a role in 5-HTR activation. None of these structures shows any variation in TM2 and TM3. These two helices are nonflexible and locked in to preserve the conserved hydrogen bonding networks.

For Class A GPCRs, several conserved inter-helical interactions, such as the TMs 1-2-7 and TMs 2-3-4 hydrogen bond networks, are present in most crystal structures. These interactions are also found in all 25 for our predicted structures for 5-HTR1E. Final 5-HTR1E structure shows conserved TM127 and TM234 network, as shown in **Supplementary Figure SK1**. It also shows TM56 salt bridge and inward rotation of Y5.58 and Y7. 43 which indicate active conformation.

**Supplementary Figure SK1.** Hydrogen bond networks of the TMs 1-2-7, TMs 2-3-4 and TMs 5-6 regions in 5HT1E receptors: (a) N^1.50^-D^2.50^-N^7.45^ hydrogen bond network, (b) S^2.45^- H^3.42^-W^4.50^ hydrogen bond network, (c) K^5.36^-E^6.30^ ionic lock for the active state. The structures shown are for top9 from SuperComBiHelix in Table 4.

**Supplementary result SK2. Relaxation of the Carboxypeptidase E (NFα1/CPE) predicted model**

Although CPN has the highest sequence identity, CPD has the same enzymatic activity (specificity for cleavage) as NFα1/CPE so CPD (PDB: 5AQ0) was used for homology template.[23] Using the previous mouse NFα1/CPE model^5^, the protein structure was relaxed through 10 ns MD, 5 ns with helix constraints and another 5 ns without any constraints. Supplementary Fig. SK2 (left) shows the protein E through MD trajectories and at 9.8 ns the structure has the lowest energy from the last 3ns trajectories. Thus, this structure in Fig. SK2 (right) was selected for 5-HTR1E docking study and truncated the last C-terminal helix which may not be involved in 5-HTR1E interaction.

**Supplementary Figure SK2**. (Left) The total, electrostatic and van der Waals (vdw) energy of the

Carboxypeptidase E (NFα1/CPE) through 10 ns molecular dynamic trajectories and (Right) the lowest energy structure of NFα1/CPE at 9.8 ns. The residues of 151-350 which is important for 5-HTR1E binding displayed in magenta. The last C-terminal helix which may not be involved in 5-HTR1E interaction was truncated at the X point.

**Supplementary result SK3. The intra SBs in EC of 5-HTR1E**

The intra SBs in EC of 5-HTR1E between R87 (EC1) & D327 (7.36) and between R161 (EC2) & D178 (EC2) were also observed (Supplementary Figure SK3 top). In IC of 5-HTR1E, the intra SBs between D119 (3. 49) & R138 (4.41) and between K210 (5.56) & E286 (6.30) were maintained (Supplementary Figure: SK3 bottom).

**Supplementary Figure SK 3**. The intra salt bridge (SB) distance in the extracellular (EC) and intracellular loops (IC) of 5-HTR1E serotonin receptor from 100 ns molecular dynamics trajectories including 5 ns with salt bridge constraints. The stable SBs are in red.

**Supplementary result SK4. Molecular docking of 5-HTR1E and NF-α1/CPE**

Fig. SK4 shows the total, electrostatic and van der Waals (vdw) interaction energies of 5-HTR1E and NFα1/CPE through 100 ns MD trajectories. Until 5ns, 4 SBs at the complex were constrained. After 5ns, the structure was relaxed without any constraints. At 45.8ns, it shows the lowest interaction E of -428.19 kcal/mol between 5-HTR1E and NFα1/CPE. But after ~53ns the interaction E keeps increasing. The initial minimized structure of 5-HTR1E and NF-α1/CPE complex shows possible interactions between the positive charged residues in the extracellular domain (EC) and negative charged residues in NFα1/CPE; K89 (EC1) and E268 (NFα1/CPE), R161 (EC2) & D306 (NFα1/CPE), R164 (EC2) & D305 (NFα1/CPE), R165 (EC2) & D304 (NFα1/CPE). However, the lowest interaction E structure from MD in Fig. SK4B revealed that only salt bridge (SB) between R161 (EC2) & D306 (NFα1/CPE) were maintained. Instead of the other SBs, two new SBs between R165 (EC2) & D251 (NFα1/CPE) and between R165 (EC2) & D342 (NF-α1/CPE) were formed. Thus, three SBs were stable; 1) R161 (EC2) & D306 (NFα1/CPE), 2) R165 (EC2) & D251 (NFα1/CPE) and 3) R165 (EC2) & E342 (NF-α1/CPE), as shown in Fig.3C (Top). In addition, the H-bonding between the backbone CO of R161 (EC2) and N345 (NFα1/CPE) was also maintained during the MD, as shown in Fig.3C (Bottom). Thus, MD confirms the strong SB interactions between 5-HTR1E and NF-α1/CPE.

**Supplementary Figure SK 4**. Molecular docking and MD studies. (A) The total, electrostatic and van der Waals (vdw) interaction energies of 5-HTR1E and Carboxypeptidase E (CPE) from 100 ns molecular dynamics trajectories (first 5 ns with salt bridge constraints). The most stable complex was found at 45.8ns. (B) The most stable interaction for Carboxypeptidase E (CPE) (blue) with the 5-HTR1E in orange from 100 ns of molecular dynamics. The main interaction is at the extracellular loop 2 of 5-HTR1E with CPE. (C) The inter salt bridge (SB) and H-bonding (HB) distance in the extracellular loops (EC) of 5-HTR1E and Carboxypeptidase E (CPE) from 100 ns molecular dynamics trajectories (first 5 ns with salt bridge constraints). The stable SBs and HBs are in red.


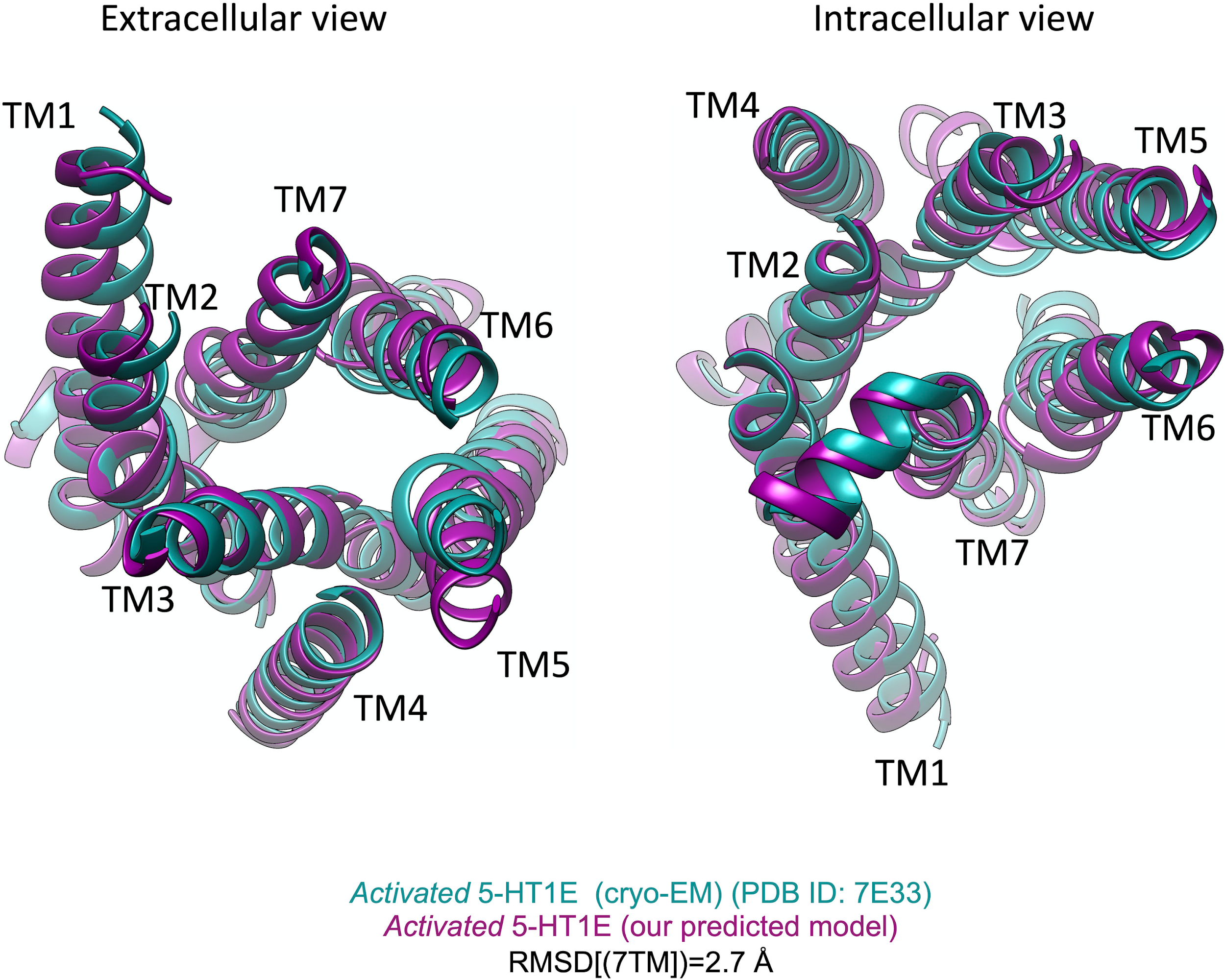


**Supplementary Figure SK5.** Comparison of our predicted active state of 5 -HT1E (in purple) with the cryo-EM structure (in green) [24].

**Design and principle of Presto-Tango assays**


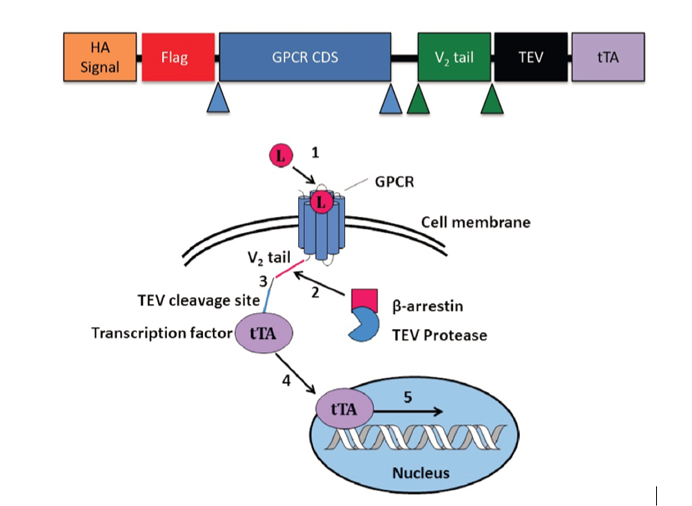


Kroeze et.al., Nat Struct Mol Biol. 2015

5-HTR1E Fold change in Presto-Tango assay

**
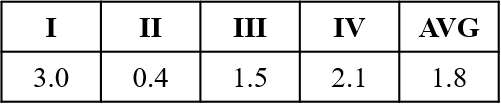
**

**Supplementary Figure S1.** High throughput Presto-Tango assay was performed by Roth lab (UNC, Chapel Hill), where a library of 324 GPCRs were screened against recombinant CPE. GPCRs with highest fold change from four experiments were selected for further screening. Out of the several target GPCRs, 5-HTR1E which is a known serotonin receptor, was found to be activated upon CPE treatment. [25]

**B**

**A**

**Supplementary Figure S2. ^125^I CPE binding to HEK 293 cell surface.**

**(A)** HEK 293 cells were incubated with different concentrations (1.25-30 nM) of ^125^I CPE (hot) with or without 500 nM cold CPE for 3h on ice in serum-free binding medium. **(B)** The specific binding was determined by measuring bound ^125^I CPE in the presence of 500 nM cold CPE.

0 nM

100 nM

10 nM

1000 nM


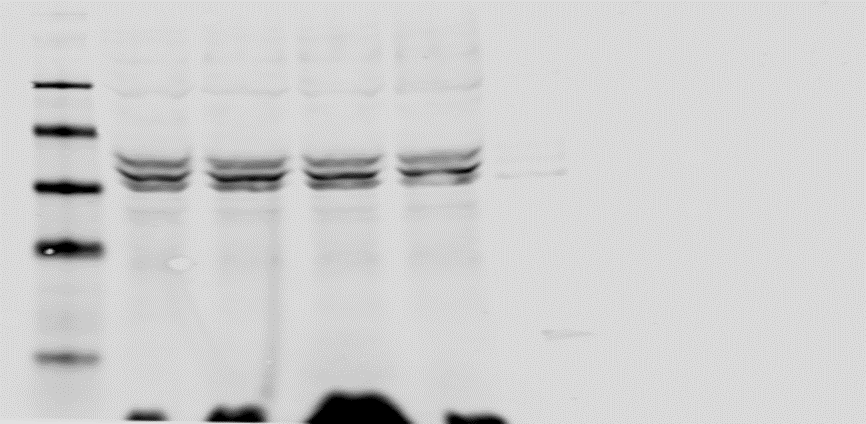

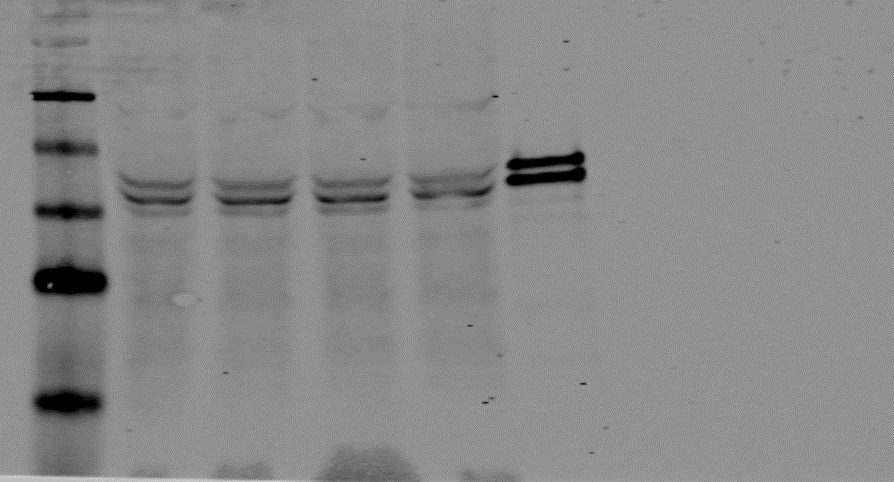


pERK

tERK

**(A1)**


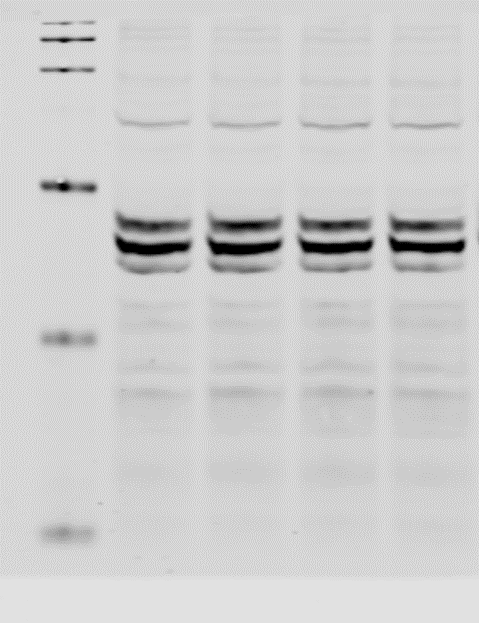


50 nM

25 nM

10 nM

0 nM


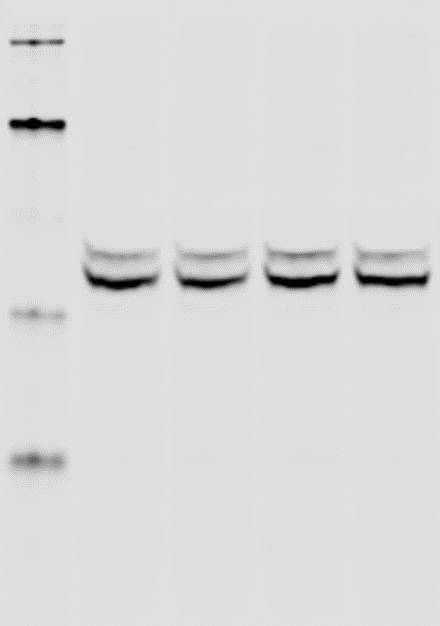


pERK

tERK

**(B1)**

**(A2)**

**(B2)**


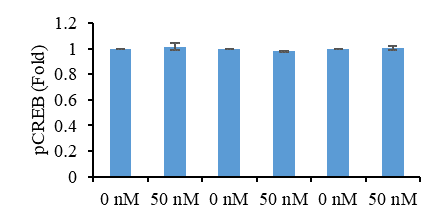


**10 min**

**20 min**

**60 min**

**(C2)**

**(C1)**

**HEK 293 (CPE)**

0 nM

0 nM

50 nM

0 nM

50 nM

50 nM

10 min

20 min

60 min


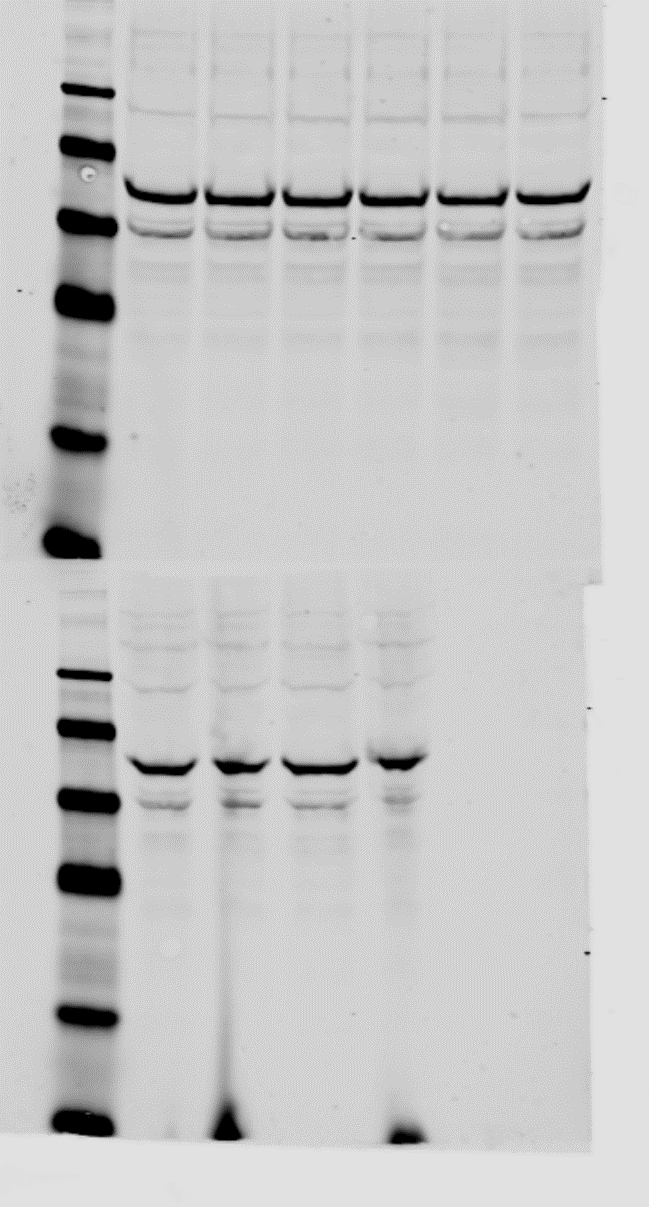

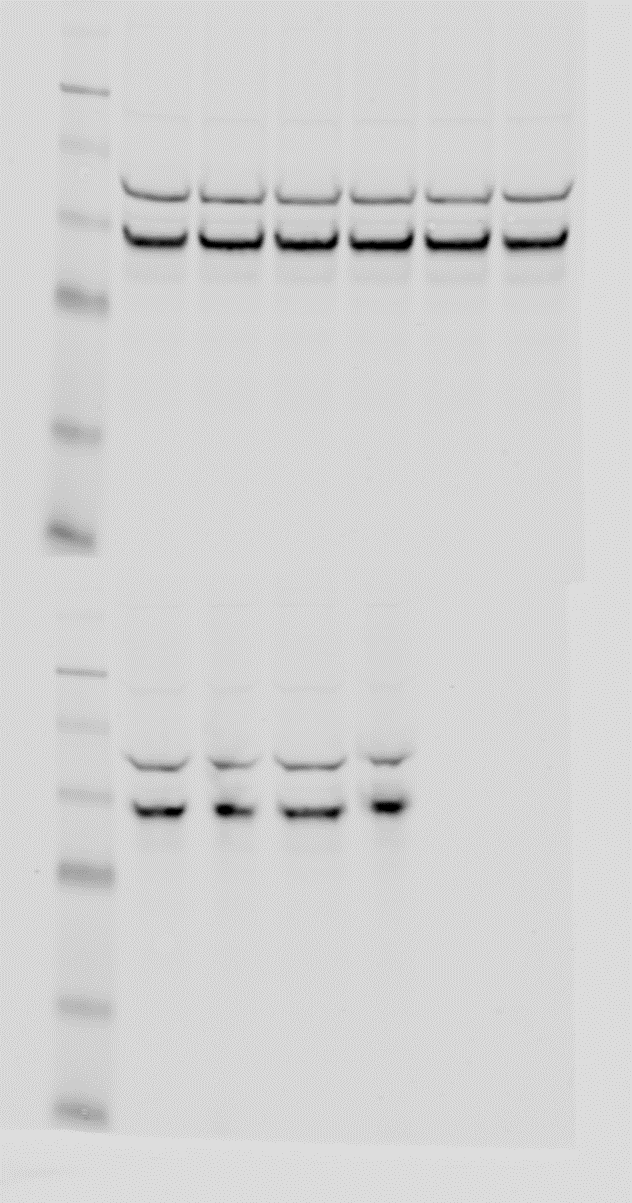


pCREB

tCREB

**Supplementary Figure S3.** Control HEK 293 cells transfected with empty vector were treated with 0 to 50 nM CPE **(A1-2)** or 1 µM 5-HT **(B1-2)** for 5 to 15 min and change in pERK 1/2 were analyzed by western blotting. Bar graphs showing the fold change in pERK1/2 after normalization with tERK1/2 as an internal control. One-way ANOVA analysis followed by Tukey’s *post hoc* multiple comparison test, [F (3, 8) = 0.8585, *ns*] N=3, for CPE and [F (3, 8) = 0.6041, *ns*] for 5-HT when compared to untreated control. **(C1-2)** control HEK 293 cells were treated with 50 nM CPE at different time points ( between 10 to 60 min) and analyzed by western blotting. Bar graphs showing the fold change in pCREB after normalization with tCREB as an internal control. Student’s t test, p>0.05 (ns) for 50 nM CPE when compared to untreated control, values are mean ± SD, N=3.

**(A) (B)**

Control

H₂O₂

H₂O₂+

5-HT


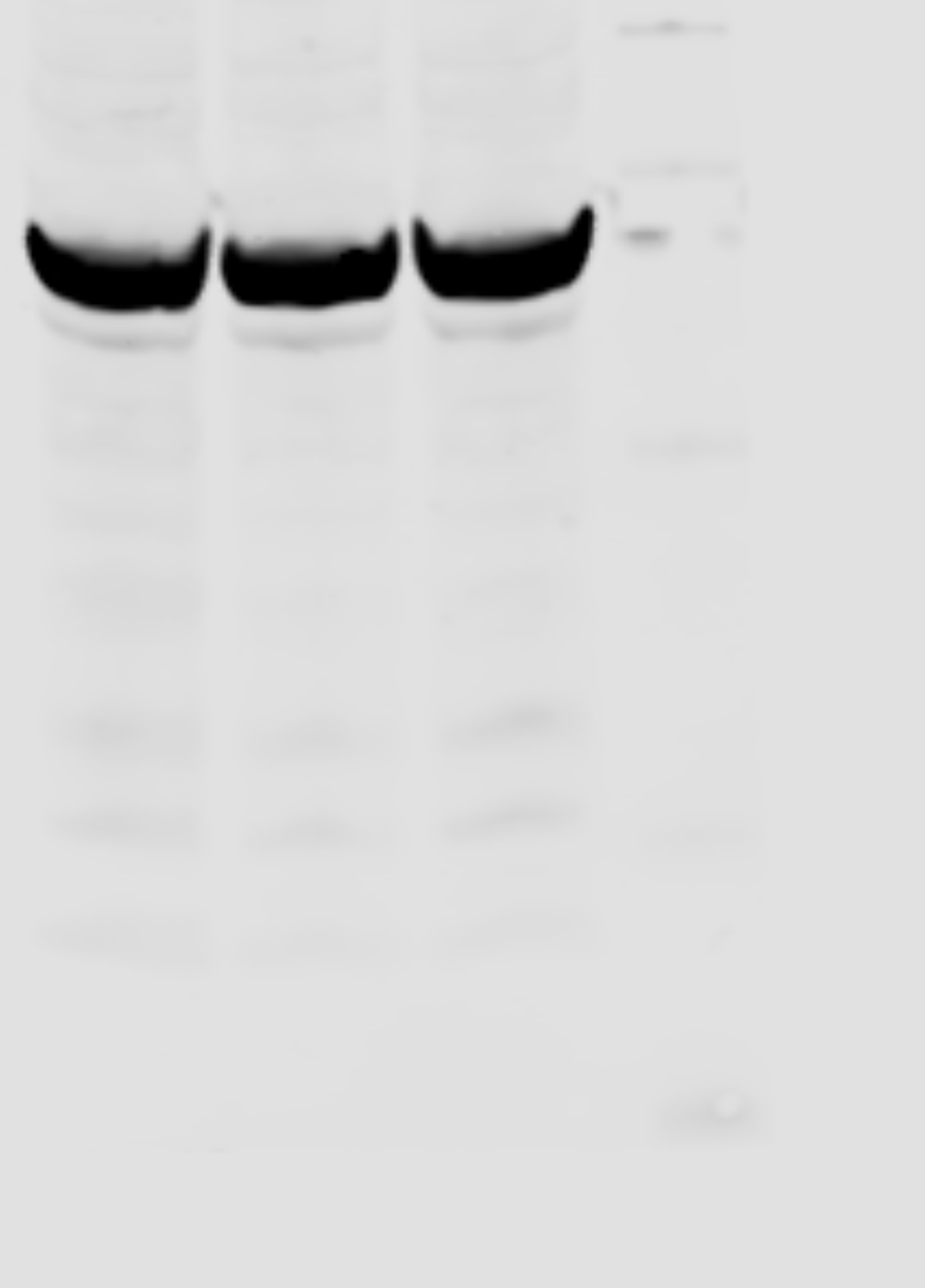

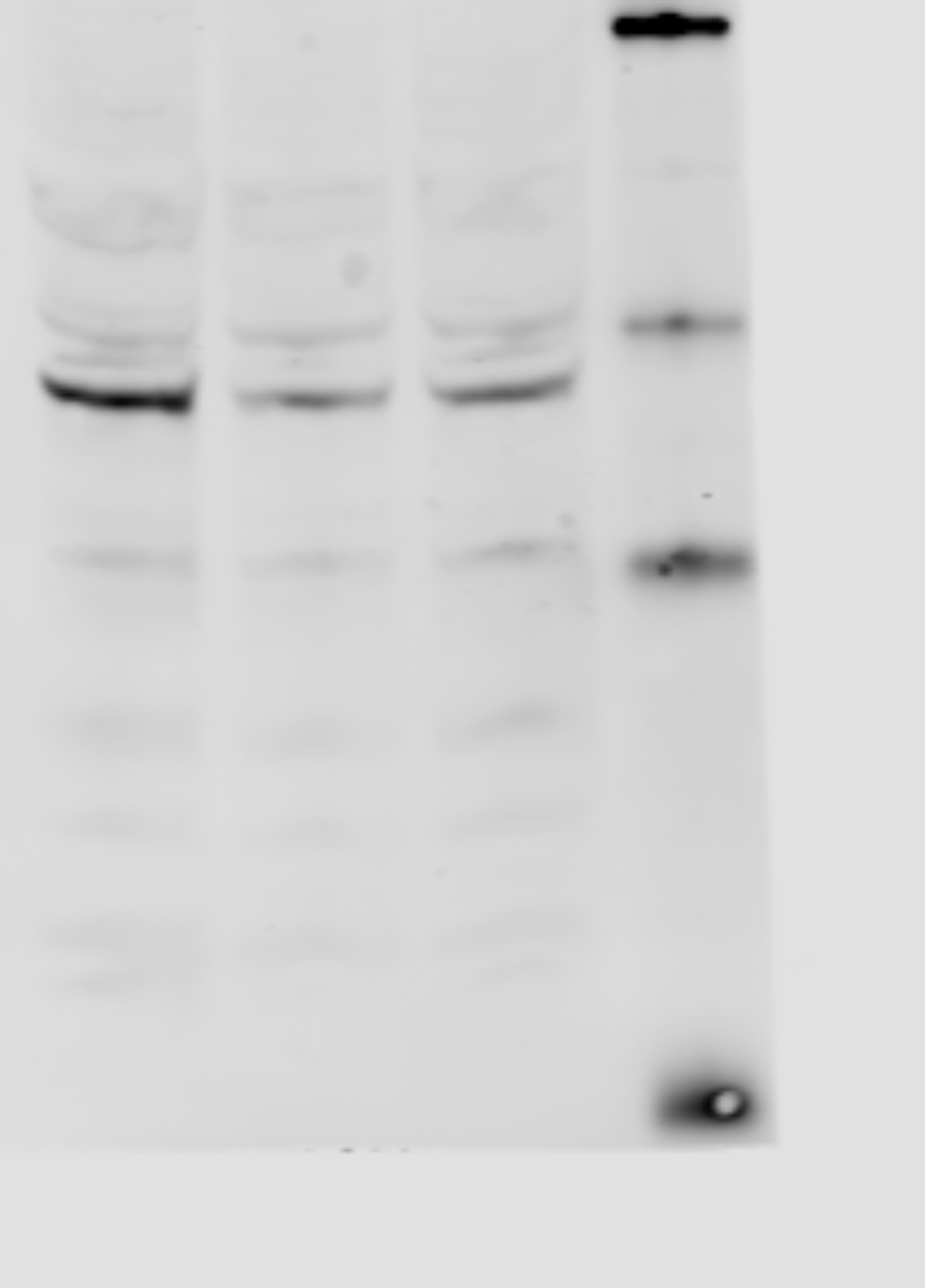


5HTR1E stables

BCL2

β-actin

**Supplementary Figure S4. Serotonin does not prevent H_2_O_2_ induced reduction in BCL2 protein level. (A1)** Western blot analysis of BCL2 protein in 5-HTR1E stable cells treated with 1 μM 5-HT for 6 h followed by 200 μM H**_2_**O**_2_** treatment overnight. **(A2)** Bar graphs showing quantification of BCL2 protein after normalization with β-actin. Results are expressed as fold change, One-way ANOVA analysis followed by Tukey’s *post hoc* multiple comparison test, F (2, 6) = 31.86 p=0.0006, controls vs H**_2_**O**_2_** *p=0.001, controls *vs* H_2_O_2_+CPE *p=0.0013, H_2_O_2_ *vs* H_2_O_2_+CPE p=*ns*, value are mean+SD, N=3,

**IHC analysis of CPE in human hippocampus**

**
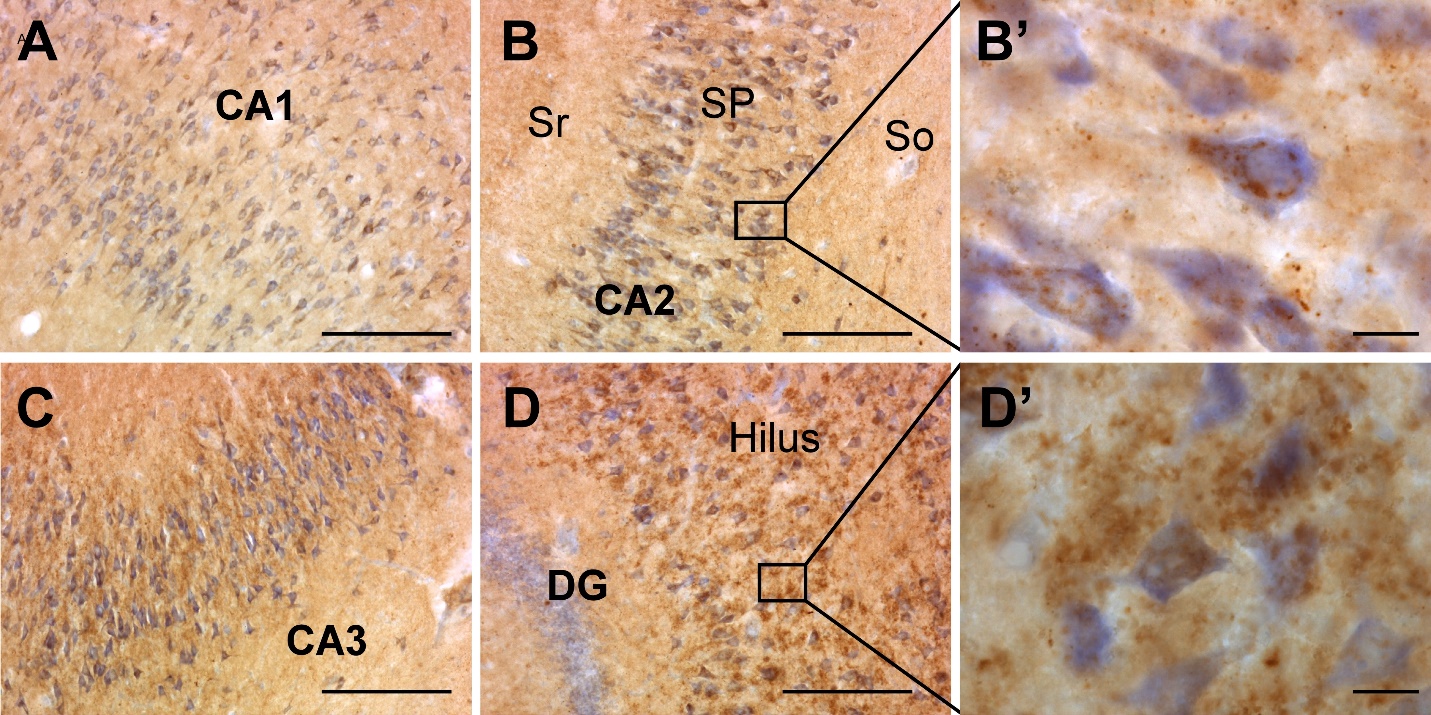
**

**Supplementary Figure S5. Immunohistochemistry for CPE**. CPE staining in human hippocampus (Bregma 22.6 mm) illustrating the widespread distribution along all hippocampal regions CA1 (A), CA2 (B), CA3 (C) and DG (D). High magnification image showing the staining within perikaria and apex of pyramidal neurons in CA2 (B’) and in DG (D’). SP (stratum pyramidale; pyramidal cell layer); Sr (stratum radiatum); So (stratum oriens). Scale bars; A-D = 500 µm, B’ and D’ = 20 µm.

**IHC analysis of 5-HTR1E in human hippocampus**

**
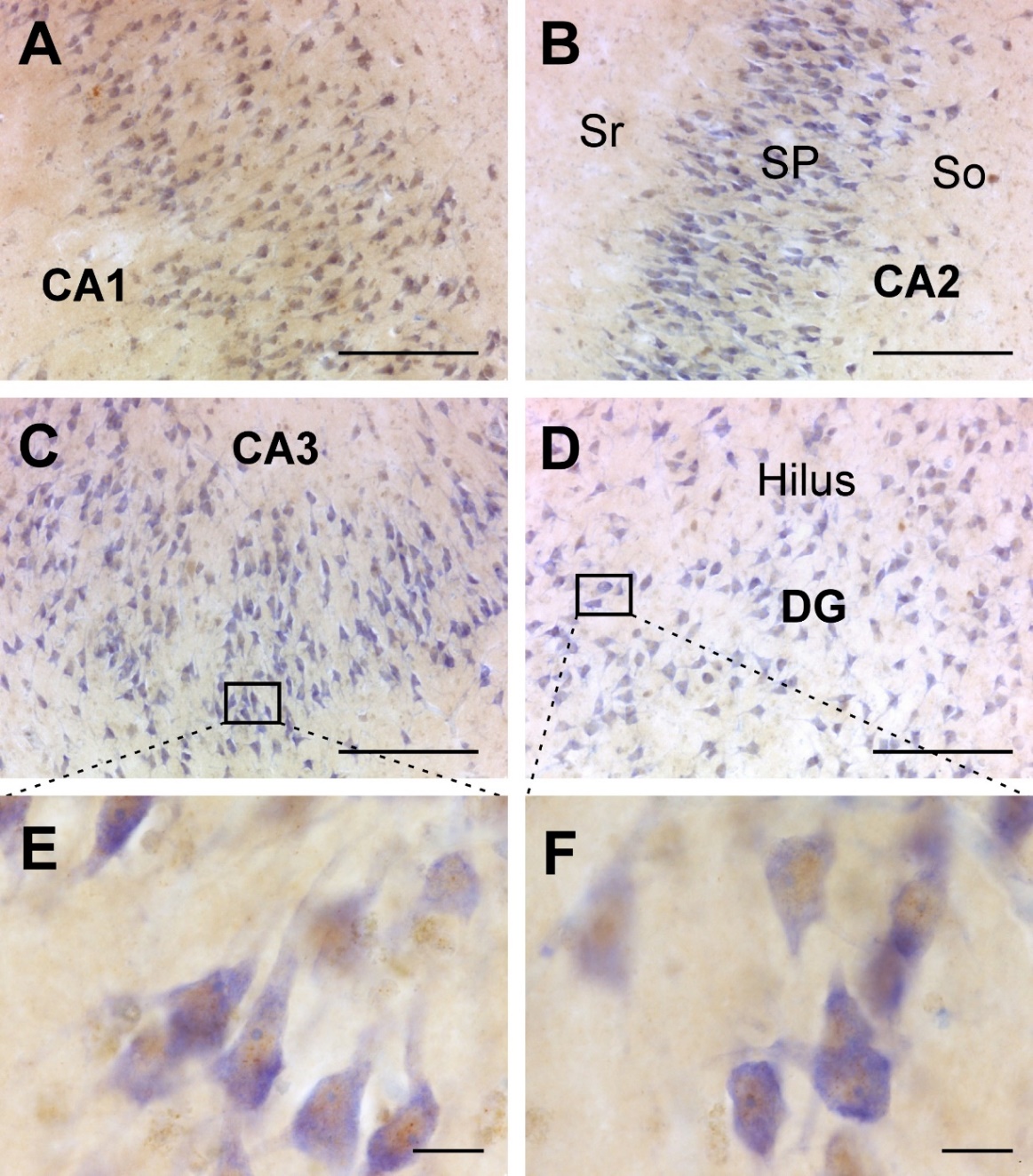
**

**Supplementary Figure S6. Immunohistochemistry for 5-HTR1E (Abnova antibody).**

5-HTR1E staining in human hippocampus (Bregma 22.6 mm) illustrating the distribution along all hippocampal regions CA1 (A), CA2 (B), CA3 (C) and DG (D). High magnification image showing the staining within perykaria in CA3 (E) and in DG (F). SP (stratum pyramidale; pyramidal cell layer); Sr (stratum radiatum); So (stratum oriens). Scale bars; A-D = 500 µm, E-F = 20 µm.

**(I) 5-HTR1E (II) CPE**


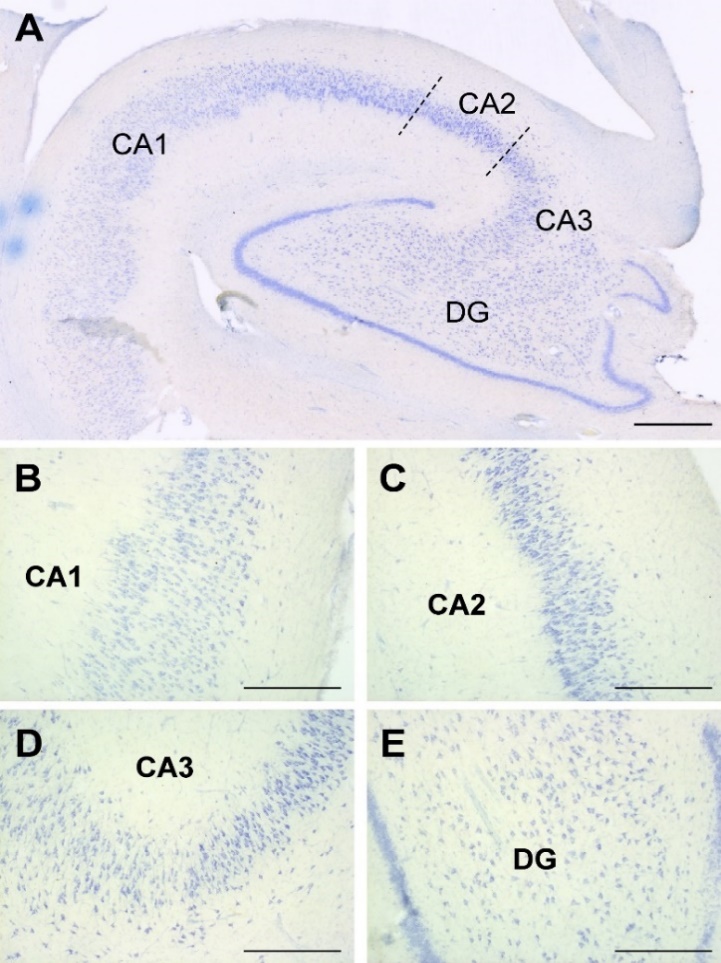

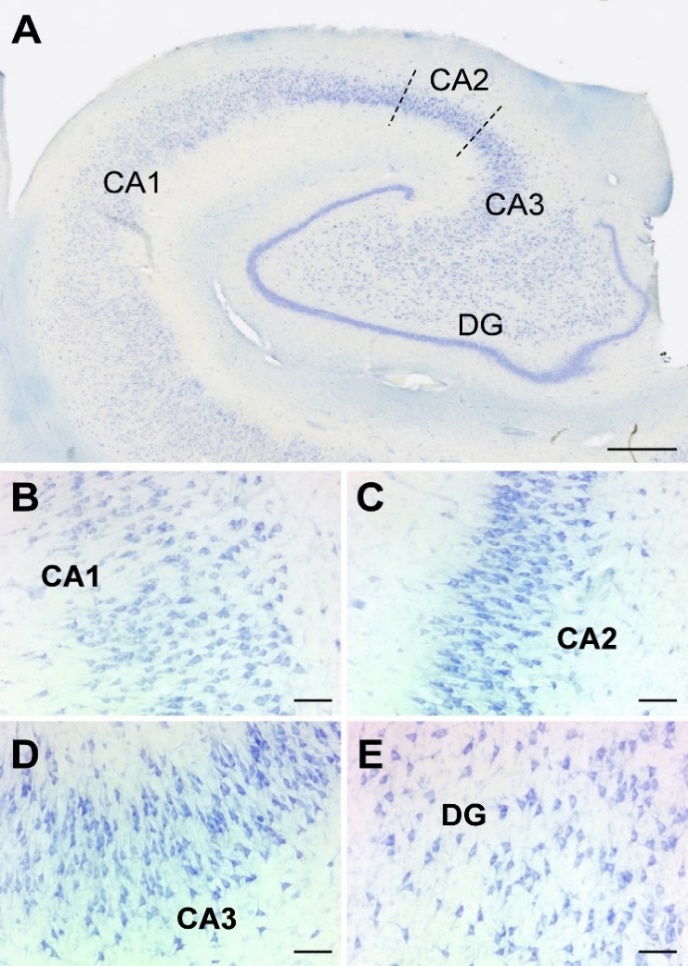


**Supplementary Figure S7. Primary antibody omission control for 5-HTR1E and CPE.**

(I) Mosaic of images at 5x of 5-HTR1E staining in human hippocampus (Bregma 22.6 mm) illustrating the widespread distribution along all hippocampal regions (A) and detail images for CA1 (B), CA2 (C), CA3 (D) and DG (E). Nonspecific staining was absent. Scale bar; A = 1000 µm, B-E = 100 µm. (II) Mosaic of images at 5x of CPE staining in human hippocampus (Bregma 22.6 mm) illustrating the widespread distribution along all hippocampal regions (A) and detail images for CA1 (B), CA2 (C), CA3 (D) and DG (E). Nonspecific staining was absent. Scale bar; A = 1000 µm, B-E = 500 µm.


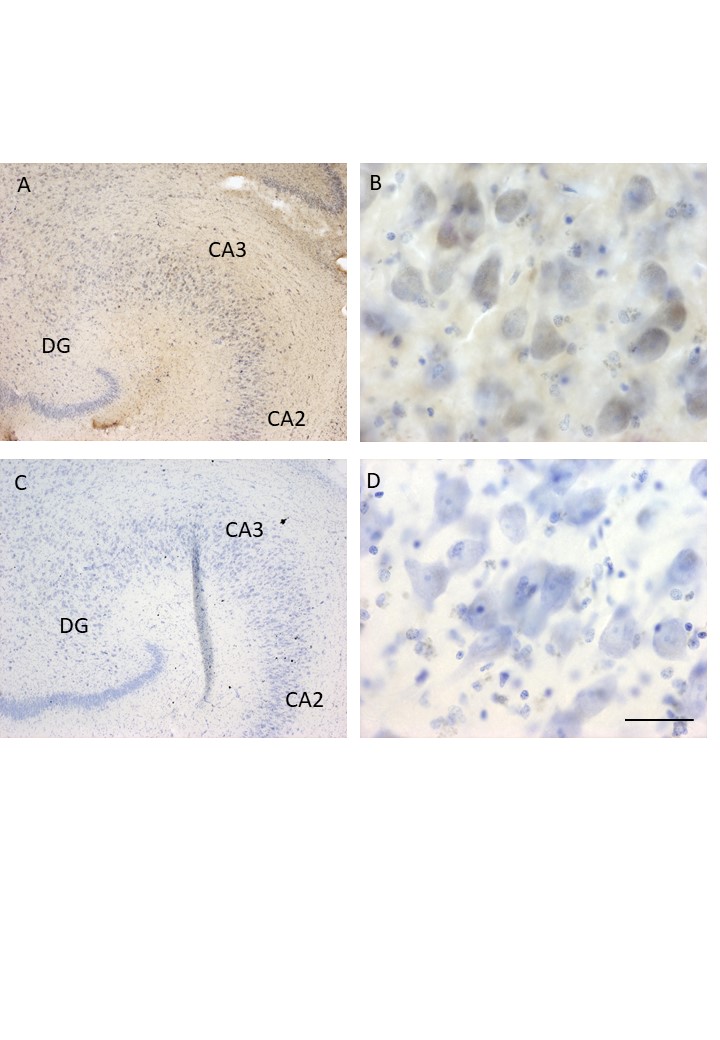


**Supplementary Figure S8. 5-HTR1E (S31 rabbit antibody) staining in human hippocampus.** In the left column low magnification images of hippocampus for S31 staining (A) and primary antibody control (C) are shown. In the right column high magnification images for CA3 region for S31 staining (B) and primary antibody control (D) are shown. Scale bar; A, C = 360 µm and B, D = 20 µm.

**Supplementary Figure S9. Primary HTR1E (Abnova) antibody absorption control**

The left column shows low magnification images of hippocampus for HTR1E staining (A), anti-HTR1E absorbed staining (C) and omission of primary antibody control (E). In the right column you can see high magnification images for CA3 region for HTR1E staining (B), anti-HTR1E absorbed staining (D) and primary antibody control (F). Note the staining is very low in both HTR1E absorbed tissue and primary antibody omission control tissues.

Scale bar; A, C, E = 220 µm and B, D, F = 20 µm.

**Supplementary video 1.** Movie of a z-stack video is composed for 23 slices at 63x magnification within 9.9 µm of thickness. The brain area corresponds to CA1.

**Supplementary video 2.** Movie of a z-stack. Reconstruction of 61 images taken each 0.37 µm across the 22.2 µm total thickness of a section. The brain area corresponds to CA2.

**Supplementary video 3.** Movie of a z-stack. Reconstruction of 27 images taken each 1 µm across the 26 µm total thickness of a section. The brain area corresponds to CA3.

**Supplementary video 4.** Movie of a z-stack. Reconstruction of 18 images taken each 1 µm across the 17 µm total thickness of a section. The brain area corresponds to DG.

**Supplementary Table S5**. Human cases used for this study (*PMD; postmortem delay before brain removal).

**
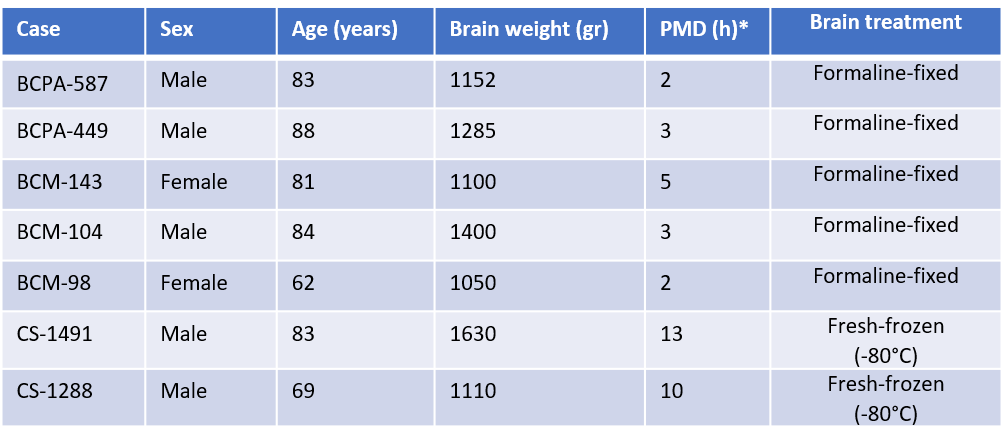
**

**Supplementary references**

1. Abrol R, Bray JK and Goddard WA, 3rd (2011) Bihelix: Towards de novo structure prediction of an ensemble of G-protein coupled receptor conformations. Proteins. doi: 10.1002/prot.23216

2. Abrol R, Griffith AR, Bray JK and Goddard WA, 3rd (2012) Structure prediction of G protein-coupled receptors and their ensemble of functionally important conformations. Methods Mol Biol 914:237-54. doi: 10.1007/978-1-62703-023-6_14

3. Bray JK, Abrol R, Goddard WA, 3rd, Trzaskowski B and Scott CE (2014) SuperBiHelix method for predicting the pleiotropic ensemble of G-protein-coupled receptor conformations. Proc Natl Acad Sci U S A 111:E72-8. doi: 10.1073/pnas.1321233111

4. Abrol R, Kim SK, Bray JK, Trzaskowski B and Goddard WA, 3rd (2013) Conformational ensemble view of G protein-coupled receptors and the effect of mutations and ligand binding. Methods Enzymol 520:31-48. doi: 10.1016/B978-0-12-391861-1.00002-2

5. Kam VWT and Goddard I, W. A. (2008) Flat-Bottom Strategy for Improved Accuracy in Protein Side-Chain Placements. J. Chem. Theory Comput. 4: 2160–2169. doi: 10.1097/COH.0b013e32830ab9dd

6. Mayo SL, Olafson BD and Goddard WA (1990) DREIDING: a generic force field for molecular simulations. J. Phys. Chem. 94:8897-8909.

7. Wang C, Jiang Y, Ma J, Wu H, Wacker D, Katritch V, Han GW, Liu W, Huang XP, Vardy E, McCorvy JD, Gao X, Zhou XE, Melcher K, Zhang C, Bai F, Yang H, Yang L, Jiang H, Roth BL, Cherezov V, Stevens RC and Xu HE ( 2013) Structural basis for molecular recognition at serotonin receptors. Sci. 340:610-4.

8. García-Nafría J, Nehmé R, Edwards PC and Tate CG (2018) Cryo-EM structure of the serotonin 5-HT1B receptor coupled to heterotrimeric Go. Nature 558, :620–623.

9. Peng Y, McCorvy JD, Harpsøe K, Lansu K, Yuan S, Popov P, Qu L, Pu M, Che T, Nikolajsen LF, Huang XP, Wu Y, Shen L, Bjørn-Yoshimoto WE, Ding K, Wacker D, Han GW, Cheng J, Katritch V, Jensen AA, Hanson MA, Zhao S, Gloriam DE, Roth BL, Stevens RC and Liu ZJ (2018) 5-HT2C Receptor Structures Reveal the Structural Basis of GPCR Polypharmacology. Cell 172:719-730.

10. Ishchenko A, Wacker D, Kapoor M, Zhang A, Han GW, Basu S, Patel N, Messerschmidt M, Weierstall U, Liu W, Katritch V, Roth BL, Stevens RC and Cherezov V (2017) Structural insights into the extracellular recognition of the human serotonin 2B receptor by an antibody. Proc Natl Acad Sci U S A. 114:8223-8228.

11. Staus DP, Hu H, Robertson MJ, Kleinhenz ALW, Wingler LM, Capel WD, Latorraca NR, Lefkowitz RJ and Skiniotis G (2020) Structure of the M2 muscarinic receptor-beta-arrestin complex in a lipid nanodisc. Nature 579:297-302. doi: 10.1038/s41586-020-1954-0

12. Sali A and Blundell TL (1993) Comparative protein modelling by satisfaction of spatial restraints. J Mol Biol 234:779-815. doi: 10.1006/jmbi.1993.1626

13. Abraham MJM, T.; Schulz, R.; Páll, S.; Smith, J. C.; Hess, B.; Lindahl, E. (2015) GROMACS: High Performance Molecular Simulations through Multi-Level Parallelism from Laptops to supercomputers. . SoftwareX 1: 19–25.

14. Huang J, Rauscher S, Nawrocki G, Ran T, Feig M, de Groot BL, Grubmuller H and MacKerell AD, Jr. (2017) CHARMM36m: an improved force field for folded and intrinsically disordered proteins. Nat Methods 14:71-73. doi: 10.1038/nmeth.4067

15. Jorgensen WL, Chandrasekhar J, Madura JD, Impey RW and Klein ML (1983) Comparison of Simple Potential Functions for Simulating Liquid Water. . J. Chem. Phys. 79:926–935.

16. Bussi G, Donadio D and Parrinello M (2007) Canonical sampling through velocity rescaling. J Chem Phys 126:014101. doi: 10.1063/1.2408420

17. Parrinello M and Rahman A (1981) Polymorphic Transitions in Single Crystals: A New Molecular Dynamics Method. . J. Appl. Phys. 52 7182–7190.

18. Essmann U, Perera L, Berkowitz ML, Darden T, Lee H and Pedersen LG (1995) A Smooth Particle Mesh Ewald Method. . J. Chem. Phys. 103: 8577–8593.

19. Miyamoto S and Kollman PA (1992) Settle: An Analytical Version of the SHAKE and RATTLE Algorithm for Rigid Water Models. . J. Comput. Chem. 13:952–962.

20. Hess B (2008) P-LINCS: A Parallel Linear Constraint Solver for Molecular Simulation. J Chem Theory Comput 4:116-22. doi: 10.1021/ct700200b

21. Stauffer W, Sheng H and Lim HN (2018) EzColocalization: An ImageJ plugin for visualizing and measuring colocalization in cells and organisms. Sci Rep 8:15764. doi: 10.1038/s41598-018-33592-8

22. Mai JM, Majtanik M and Paxinos G (2015) Atlas of the Human Brain. 4th edition. Academic Press:456.

23. Dhanvantari S, Arnaoutova I, Snell CR, Steinbach PJ, Hammond K, Caputo GA, London E and Loh YP (2002) Carboxypeptidase E, a prohormone sorting receptor, is anchored to secretory granules via a C-terminal transmembrane insertion. Biochem. 41:52-60.

24. Xu P, Huang S, Zhang H, Mao C, Zhou XE, Cheng X, Simon IA, Shen DD, Yen HY, Robinson CV, Harpsoe K, Svensson B, Guo J, Jiang H, Gloriam DE, Melcher K, Jiang Y, Zhang Y and Xu HE (2021) Structural insights into the lipid and ligand regulation of serotonin receptors. Nature 592:469-473. doi: 10.1038/s41586-021-03376-8

25. Kroeze WK, Sassano MF, Huang XP, Lansu K, McCorvy JD, Giguere PM, Sciaky N and Roth BL (2015) PRESTO-Tango as an open-source resource for interrogation of the druggable human GPCRome. Nat Struct Mol Biol 22:362-9. doi: 10.1038/nsmb.3014
